# Lesion Site‐Targeted Microspheres Modulate Nav1.7‐Related Signaling for Osteoarthritis Treatment

**DOI:** 10.1002/advs.75986

**Published:** 2026-06-04

**Authors:** Cheng Chen, Jiaying Li, Jinjin Ma, Xiaonan Yuan, Yao Xiao, Yang Zhang, Yuan Chen, Hao Jiang, Hui He, Jie Hu, Qianglong Chen, Jinniu Zhang, Bin Li, Yun Zhou, Fengxuan Han, Yong Wang

**Affiliations:** ^1^ Department of Orthopedic Surgery The Affiliated Yixing Hospital of Jiangsu University Wuxi Jiangsu China; ^2^ Medical 3D Printing Center Orthopedic Institute Department of Orthopedic Surgery The First Affiliated Hospital School of Basic Medical Sciences Interdisciplinary Innovation Center for Nanomedicine MOE Key Laboratory of Geriatric Diseases and Immunology Changzhou Geriatric Hospital Suzhou Medical College Biomedical Basic Research Center of Jiangsu Soochow University Suzhou Jiangsu China; ^3^ Regenerative Medicine & Tissue Engineering Research Center Institute of Medical Innovation and Translation The Affiliated Yixing Hospital of Jiangsu University Yixing China; ^4^ Department of Rehabilitation Medicine The Second Affiliated Hospital of Anhui Medical University Hefei Anhui China

**Keywords:** acid‐responsive delivery, carbamazepine, Nav1.7, osteoarthritis, type II collagen‐targeting peptide

## Abstract

Osteoarthritis (OA) progression involves cartilage degeneration, inflammation, and pain. Recent evidence suggests that the voltage‐gated sodium channel Nav1.7, encoded by *Scn9a*, links nociceptive signaling with chondrocyte metabolic regulation. Here, we developed an acid‐responsive, cartilage‐targeted delivery system based on WYRGRL peptide‐modified chondroitin sulfate/oxidized hyaluronan composite microspheres loaded with carbamazepine (CBZ), termed CBZ/WCOM, for sustained intra‐articular CBZ delivery. In interleukin‐1β (IL‐1β)–challenged chondrocytes, CBZ/WCOM attenuated inflammatory and catabolic responses, as indicated by reduced *Ptgs2*, *Mmp13*, and *Adamts5* expression, while partially restoring anabolic markers including *Col2a1* and *Acan*. Mechanistically, CBZ/WCOM reduced sodium current density, modulated Na⁺/Ca²⁺ exchanger (NCX)‐associated Ca^2^^+^ dynamics, and increased heat shock protein 70 (HSP70) and Midkine secretion, supporting the involvement of Nav1.7‐related sodium channel signaling in chondrocyte regulation. In a mouse destabilization of the medial meniscus (DMM) model, intra‐articular CBZ/WCOM improved mechanical withdrawal thresholds, reduced pain‐related neural remodeling, decreased osteophyte formation, partially preserved joint‐space width, improved cartilage matrix phenotype, and ameliorated subchondral bone alterations. By integrating WYRGRL‐mediated type II collagen (COL2) targeting with pH‐triggered CBZ release, CBZ/WCOM supports lesion‐localized drug retention and provides a minimally invasive strategy for local OA therapy.

## Graphical abstract

Cartilage‐targeted carbamazepine‐loaded WYRGRL‐modified composite microspheres (CBZ/WCOM) anchor to exposed type II collagen in osteoarthritic lesions and release carbamazepine under acidic conditions. This bind‐then‐release platform modulates Nav1.7‐related sodium signaling, Na⁺/Ca²⁺ exchanger‐associated Ca^2^^+^ dynamics, and heat shock protein 70/Midkine responses, thereby attenuating inflammation, matrix catabolism, and pain‐related remodeling while supporting cartilage protection.

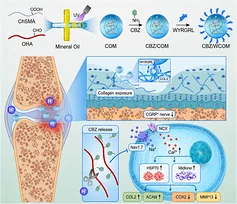

## 1 Introduction

Osteoarthritis (OA) is one of the most common joint diseases, affecting over 500 million people worldwide and rising in prevalence with global population aging [1]. As a chronic, degenerative disease, OA is characterized by disrupted chondrocyte homeostasis with progressive loss of cartilage extracellular matrix, accompanied by synovial inflammation and pathological remodeling of the subchondral bone [2, 3]. Pain is not only the dominant clinical complaint but also a driver of disease progression [4, 5]. Inflammatory mediators and neuropeptides sensitize peripheral afferents and amplify central nociceptive processing, while matrix‐degradation fragments further activate nociceptors, forming an “inflammation–pain–degeneration” feed‐forward loop [6, 7]. Recent studies have revealed that the voltage‐gated sodium channel Nav1.7 is also expressed in chondrocytes and has been implicated in chondrocyte metabolic regulation, thereby influencing OA progression [8]. This discovery establishes a mechanistic link between nociceptive signaling and chondrocyte metabolic regulation, and it inspires the development of integrated therapeutic strategies targeting the ‘metabolism–pain axis’ [9, 10].

During OA progression, inflammatory mediators released from chondrocytes, synovial cells, and subchondral bone cells jointly contribute to cartilage matrix degradation, subchondral bone remodeling, and pain development [3, 10, 11]. Pro‐inflammatory cytokines such as interleukin‐1β (IL‐1β) and tumor necrosis factor‐α (TNF‐α) disrupt chondrocyte homeostasis by promoting catabolic enzymes, including matrix metalloproteinases (MMPs) and a disintegrin and metalloproteinase with thrombospondin motifs (ADAMTSs), while suppressing anabolic matrix components such as type II collagen (COL2) and Aggrecan [10]. Inflammatory mediators such as cyclooxygenase‐2 (COX2)‐derived prostanoids further amplify local inflammation and sensitize peripheral nociceptors, thereby linking cartilage inflammation to OA‐associated pain [4]. In parallel, matrix‐degradation products and subchondral bone remodeling can promote aberrant sensory nerve remodeling within the osteochondral unit [6, 13]. This interaction among inflammation, matrix catabolism, subchondral remodeling, and pain‐related innervation forms a feed‐forward loop that accelerates OA progression and contributes to chronic pain [11, 12]. Therefore, therapeutic strategies capable of simultaneously modulating inflammatory‐catabolic signaling and pain‐related neural responses may provide broader benefits for OA treatment.

Current OA management is increasingly transitioning from symptomatic pain relief toward disease‐modifying strategies [1, 2]. The voltage‐gated sodium channel isoform Nav1.7, a critical mediator of nociceptive transmission, has emerged as a promising non‐opioid analgesic target [10, 11, 12, 13]. Although the clinical outcomes of highly selective Nav1.7 inhibitors remain inconsistent, convergent genetic and pharmacological evidence underscores its central role in pain modulation [14, 15]. Loss‐of‐function mutations in *Scn9a* (encoding Nav1.7) cause congenital insensitivity to pain, whereas gain‐of‐function variants result in paroxysmal extreme pain disorder that is notably responsive to carbamazepine (CBZ) [16, 17]. In preclinical models, selective ablation of Nav1.7 in sensory neurons markedly attenuates both inflammatory and neuropathic pain [15, 18]. CBZ, an anticonvulsant with repurposing potential, acts as a broad voltage‐gated sodium channel blocker and reduces pathological neuronal hyperexcitability by stabilizing sodium channels in their inactivated state. Although achieving an optimal balance between selectivity and efficacy remains challenging for Nav1.7‐targeted therapies, intra‐articular, cartilage‐targeted delivery of CBZ may increase local drug exposure at cartilage lesions while reducing systemic exposure [19]. This strategy may help alleviate OA‐associated pain‐related responses and improve chondrocyte metabolic imbalance linked to Nav1.7‐related sodium channel signaling.

During OA progression, the joint cavity develops a mildly acidic and protease‐enriched microenvironment. Cartilage degradation and synovial inflammation disrupt normal cellular metabolism, resulting in increased glycolytic activity and lactate accumulation that collectively lower synovial fluid pH [20]. In parallel, persistent inflammation upregulates the activation of catabolic enzymes, such as matrix metalloproteinases (MMPs) and aggrecanases (ADAMTSs), which accelerate extracellular matrix decomposition [21, 22, 23]. Moreover, the normal articular cartilage matrix is shielded by an intact superficial layer in which type II collagen (COL2) fibers are embedded within a dense proteoglycan network. After OA, however, mechanical overloading, enzymatic degradation, and inflammatory insults disrupt this protective barrier, leading to the pathological exposure of COL2 on the cartilage surface and thus providing a disease‐specific and highly accessible molecular target for OA treatment [24, 25, 26]. Previous studies have reported that the COL2‐binding peptide WYRGRL can selectively recognize and anchor to damaged cartilage regions, thereby guiding therapeutic carriers toward lesional sites [25].

**SCHEME 1:**
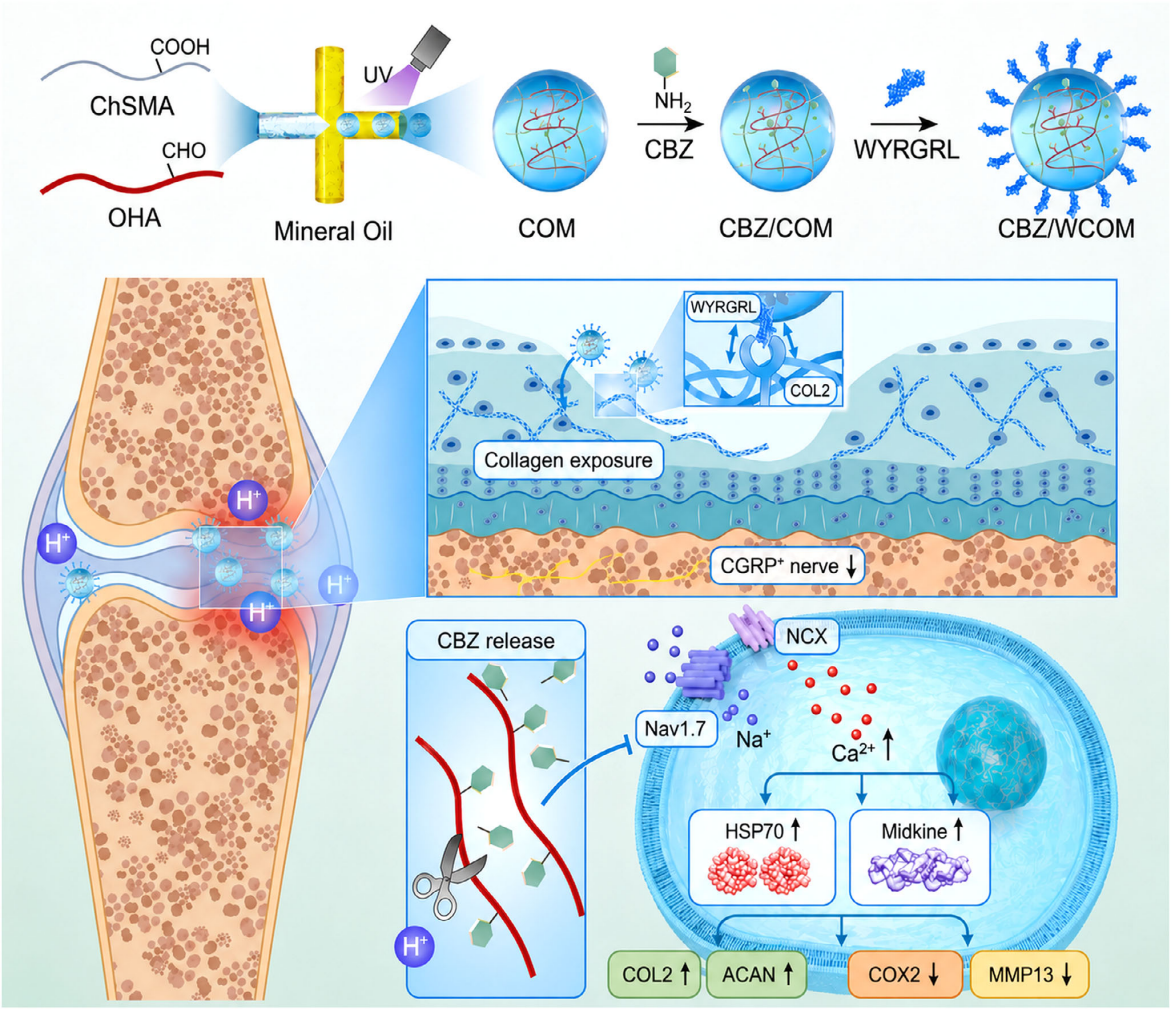
Schematic illustration of cartilage‐targeting, acid‐responsive CBZ/WCOM microspheres for intra‐articular osteoarthritis therapy. Microspheres were fabricated by microfluidics from methacrylated chondroitin sulfate and oxidized hyaluronic acid to form composite microspheres (COM), followed by carbamazepine (CBZ) loading and conjugation with the type II collagen‐binding peptide WYRGRL to obtain CBZ/WCOM. After intra‐articular injection, CBZ/WCOM preferentially anchors to exposed type II collagen (COL2) at damaged cartilage sites through WYRGRL‐mediated recognition, while the acidic osteoarthritic microenvironment promotes CBZ release through acid‐labile linkage cleavage. The released CBZ modulates sodium channel activity and Nav1.7‐related signaling, regulates Na⁺/Ca²⁺ exchanger (NCX)‐associated Ca^2^^+^ dynamics, and promotes heat shock protein 70 (HSP70)/Midkine‐related downstream responses, thereby reducing inflammatory and catabolic signaling while preserving cartilage matrix homeostasis.

In this study, based on the pathophysiological features of OA, we engineered an acid‐responsive, cartilage‐targeted composite microsphere platform for intra‐articular CBZ delivery. The overall design and therapeutic mechanism of CBZ/WCOM microspheres are illustrated in Scheme 1. CBZ/WCOM microspheres were fabricated by microfluidics integration of chondroitin sulfate (ChS) and oxidized hyaluronic acid (OHA), followed by CBZ loading and surface conjugation with the COL2‐binding peptide WYRGRL via 1‐ethyl‐3‐(3‐dimethylaminopropyl)carbodiimide/N‐hydroxysuccinimide (EDC/NHS)‐mediated coupling. After intra‐articular injection, CBZ/WCOM is designed to preferentially anchor to damaged cartilage regions through WYRGRL‐mediated recognition of exposed COL2. CBZ/WCOM then releases CBZ in response to the mildly acidic OA microenvironment, thereby modulating Nav1.7‐related sodium channel activity, attenuating inflammatory and catabolic signaling, supporting cartilage matrix homeostasis, and alleviating OA‐associated pain‐related responses. Together, this study aimed to design, characterize, and validate CBZ/WCOM as a targeted intra‐articular delivery platform, providing a multimodal local therapeutic strategy for OA‐associated pain and structural degeneration.

## 2 Results

### 2.1 Verification of Carbamazepine for Chondrocyte Metabolism Modulation

**FIGURE 1:**
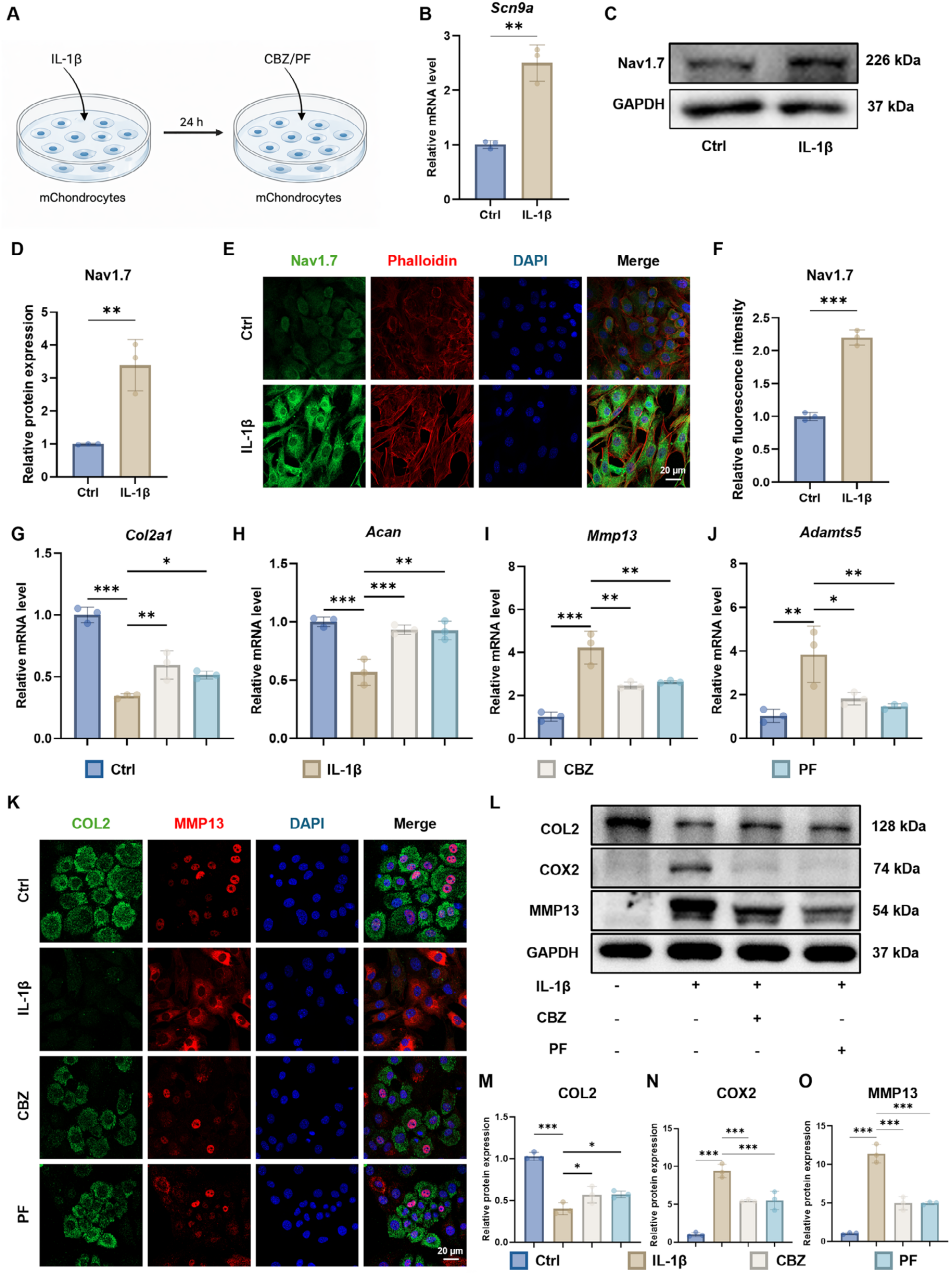
Evaluation of Nav1.7 involvement in CBZ‐mediated modulation of chondrocyte metabolism under inflammatory conditions. (A) Schematic illustration of the in vitro experimental design in which mouse chondrocytes were stimulated with IL‐1β and subsequently treated with CBZ or PF. (B) RT‐qPCR analysis of Scn9a in Ctrl and IL‐1β groups. (C) Western blot analysis of Nav1.7 protein expression in Ctrl and IL‐1β groups. (D) Quantification of Nav1.7 protein expression from (C) by densitometric analysis. (E) Immunofluorescence staining of Nav1.7 (green) co‐labeled with F‐actin (phalloidin, red) and nuclei (DAPI, blue) in chondrocytes. (F) Quantification of Nav1.7 fluorescence intensity in the indicated groups. (G–J) RT‐qPCR analysis of *Col2a1*, *Acan*, *Mmp13*, and *Adamts5* mRNA levels in Ctrl, IL‐1β, CBZ, and PF groups. (K) Immunofluorescence co‐staining of COL2 (green) and MMP13 (red) with DAPI (blue) in the indicated groups. (L) Western blot analysis of COL2, MMP13, and COX2 expression in Ctrl, IL‐1β, CBZ, and PF groups. (M–O) Quantification of protein expression. Data are presented as mean ± SD; n = 3. ^*^ *p* < 0.05; ^**^ *p* < 0.01; ^***^ *p* < 0.001.

Primary mouse chondrocytes were stimulated with IL‐1β for 24 h to establish an in vitro inflammatory model, as illustrated in Figure 1A. RT‐qPCR, western blot, and immunofluorescence analyses consistently showed that IL‐1β markedly increased Nav1.7 expression in chondrocytes (Figure 1B–F). Cell Counting Kit‐8 (CCK‐8) assay further showed that CBZ maintained good cell viability within the tested concentration range, and 10 µm was selected for subsequent experiments (Figure S1A). PF‐04856264 (PF), a selective Nav1.7 inhibitor, was included as a pharmacological control at a concentration of 1 µm. Quantitative real‐time PCR (RT‐qPCR) analysis showed that IL‐1β significantly decreased the expression of the anabolic genes *Col2a1* and *Acan* and increased the expression of the catabolic genes *Mmp13* and *Adamts5*; these changes were partially reversed by both CBZ and PF (Figure 1G–J). In addition, the IL‐1β‐induced upregulation of the inflammatory genes *Nos2* and *Ptgs2* was also attenuated by CBZ and PF (Figure S1B,C). Consistently, immunofluorescence staining showed reduced COL2 and increased matrix metalloproteinase 13 (MMP13) signals after IL‐1β stimulation, whereas both CBZ and PF partially restored these changes (Figure 1K and Figure S1D,E). Western blot analysis further confirmed that IL‐1β decreased COL2 protein expression and increased MMP13 and COX2 levels, while treatment with CBZ or PF partially reversed these alterations (Figure 1L–O).

### 2.2 Microsphere Fabrication and Characterizations

**FIGURE 2:**
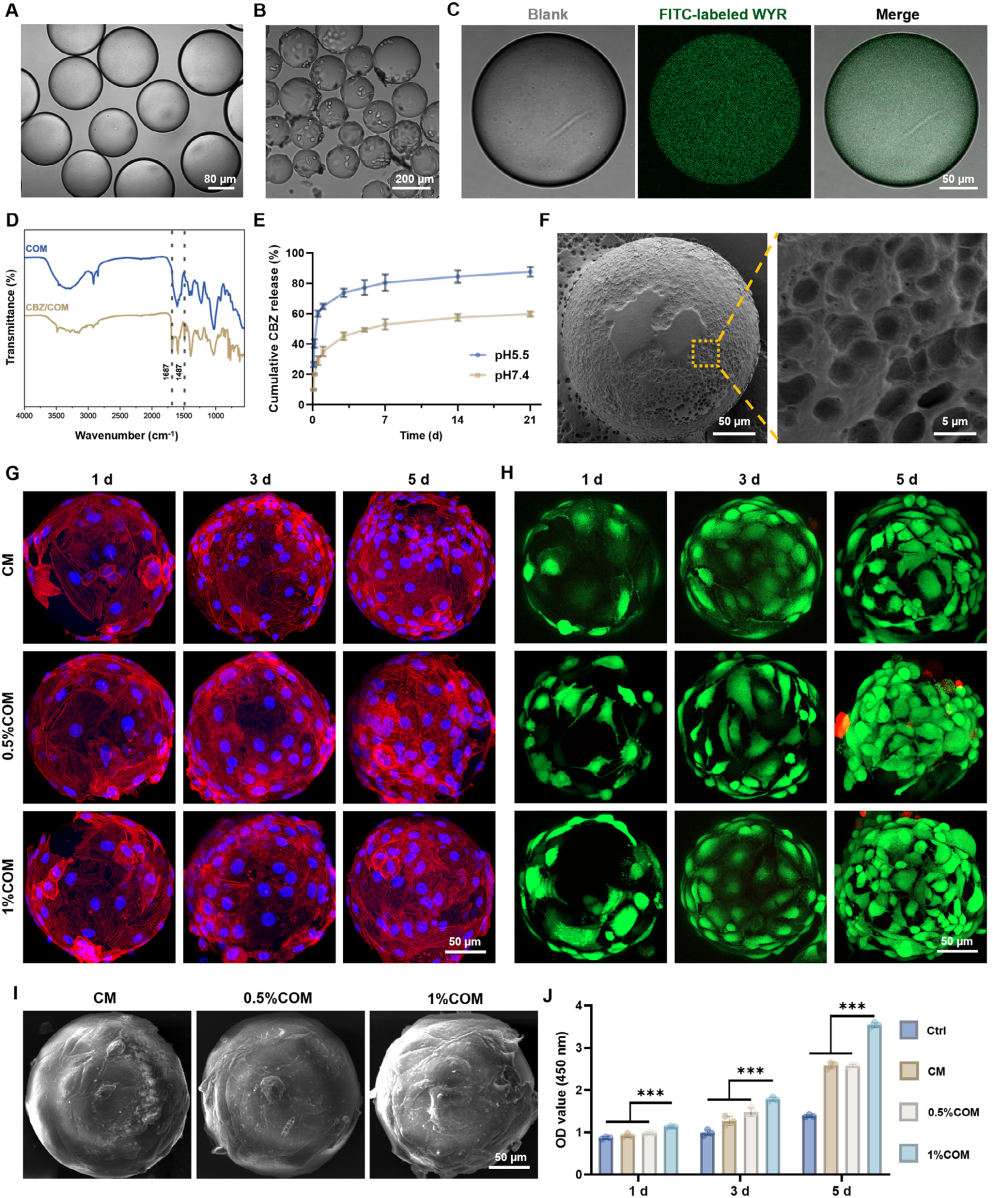
Characterizations and biocompatibility evaluation of microspheres. (A) Bright‐field image of composite microspheres (COM). (B) Low‐magnification image of microspheres after cell seeding on their surfaces. (C) Fluorescence image of COL2–binding peptide (WYRGRL)‐labeled microspheres. (D) FTIR spectra of COM and CBZ/COM. (E) Cumulative CBZ release profiles. (F) SEM images of a representative microsphere (left) and its internal structure (right). (G) Fluorescence staining of chondrocyte cytoskeleton (phalloidin, red) and nuclei (DAPI, blue) cultured on ChS‐based microspheres (CM), 0.5% COM, and 1% COM for 1, 3, and 5 days. (H) Live/dead staining of chondrocytes on ChS‐based microspheres (CM), 0.5% COM, and 1% COM for 1, 3, and 5 days (Calcein‐AM, green; EthD‐1/PI, red). (I) SEM images of cell‐laden CM, 0.5% COM, and 1% COM after 5 days of culture. (J) Cell viability determination of chondrocytes by CCK‐8 assay cultured with CM, 0.5% COM, and 1% COM for 1, 3, and 5 days. Data are presented as mean ± SD; *n* = 3. ^***^ *p* < 0.001.

OHA was synthesized following established protocols [27]. Fourier transform infrared spectroscopy (FTIR) revealed a new band at 1735 cm^−^^1^ corresponding to C═O stretching, confirming successful fabrication of OHA (Figure S2A). ChS and OHA were then co‐assembled by microfluidics to yield composite microspheres (COM) (Figure 2A). Bright‐field images and size analysis demonstrated uniform particle distribution with a mean diameter of ∼150 µm (Figure S2B). In enzyme‐containing phosphate‐buffered saline (PBS) with hyaluronidase and type II collagenase, the microspheres underwent complete degradation within 24 days, demonstrating favorable in vitro degradability (Figure S2C). CBZ/COM was obtained after immersing COM in the CBZ solution. Compared with COM, CBZ/COM displayed a new absorption band at 1687 cm^−^^1^, indicating imine (Schiff‐base, C═N) formation (Figure 2D). Finally, fluorescein isothiocyanate (FITC)‐labeled COL2‐targeting peptide (WYRGRL) was grafted to the microsphere surface by EDC/NHS coupling to generate CBZ/WCOM. Confocal microscopy images showed strong colocalization of FITC signal with microsphere contours, confirming successful WYRGRL conjugation (Figure 2C). To determine the biocompatibility, mouse chondrocytes were seeded on composite microspheres with different OHA ratios. After seeding, primary chondrocytes adhered to the microsphere surfaces, as shown in the low‐magnification images (Figure 2B). After 5 days of culture, scanning electron microscopy (SEM) further showed cell‐laden microspheres in all groups (Figure 2I). However, 1% COM showed better capacity for the promotion of cell proliferation, and the number of chondrocytes on 1% COM was higher than that on CM and 0.5% COM after 5 days of culture, indicating the incorporation of OHA improved the cytocompatibility of the microspheres (Figure 2G,H,J). In contrast, increasing the OHA ratio decreased the Young's modulus of the hydrogels, as shown by the stress–strain curves and Young's modulus analysis (Figure S2D,E). In preliminary experiments, formulations with OHA proportions exceeding 1% were difficult to form into stable microspheres. Therefore, 1% COM was selected for subsequent experiments. CBZ release behavior was measured by UV–Vis quantification. CBZ/WCOM released more CBZ under acidic conditions than under physiological pH, indicating pH‐responsive release behavior (Figure 2E).

### 2.3 CBZ‐Loaded Microspheres Regulate Inflammatory Responses and Matrix Metabolism in IL‐1β‐Stimulated Chondrocytes

**FIGURE 3:**
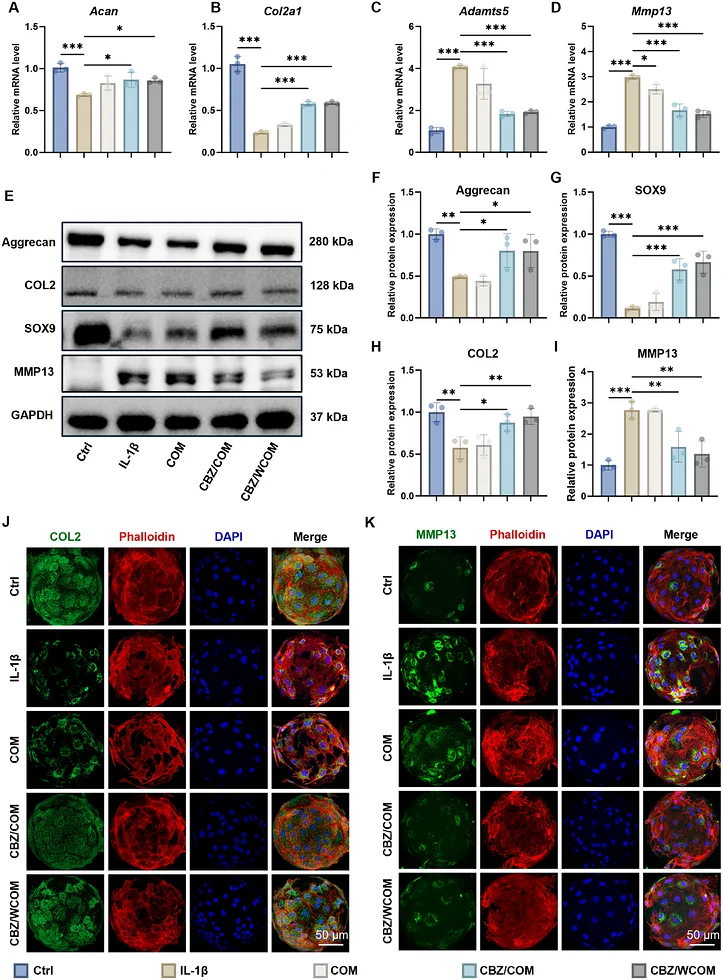
Evaluation of microsphere‐mediated regulation of chondrocyte metabolism under inflammatory conditions. (A–D) RT‐qPCR analysis of *Col2a1*, *Acan*, *Mmp13*, and *Adamts5* mRNA levels in Ctrl, IL‐1β, COM, CBZ/COM, and CBZ/WCOM groups. (E) Western blot analysis of Aggrecan, COL2, SOX9, and MMP13 expression in the indicated groups. (F–I) Quantification of protein expression from (E) by densitometric analysis. (J) Immunofluorescence staining of COL2 (green) co‐labeled with F‐actin (phalloidin, red) and nuclei (DAPI, blue) in chondrocyte spheroids. (K) Immunofluorescence staining of MMP13 (green) co‐labeled with F‐actin (phalloidin, red) and nuclei (DAPI, blue) in chondrocyte spheroids. Data are presented as mean ± SD; *n* = 3. ^*^ *p* < 0.05; ^**^ *p* < 0.01; ^***^ *p* < 0.001.

In the in vitro inflammatory chondrocyte model, IL‐1β stimulation markedly disturbed cartilage matrix metabolism. RT‐qPCR analysis showed that IL‐1β significantly decreased the expression of the anabolic genes *Col2a1* and *Acan* and increased the expression of the catabolic genes *Mmp13* and *Adamts5* (Figure 3A–D). Compared with the IL‐1β group, the COM group showed limited effects, whereas both the CBZ/COM and CBZ/WCOM groups partially restored *Col2a1* and *Acan* expression and reduced *Mmp13* and *Adamts5* expression. Consistently, western blot showed that IL‐1β reduced COL2, SOX9 and Aggrecan protein levels, and increased MMP13 expression. These changes were only marginally affected by COM but were partially reversed in both the CBZ/COM and CBZ/WCOM groups (Figure 3E–I). Immunofluorescence staining of chondrocyte spheroids further confirmed these findings, showing decreased COL2 fluorescence and increased MMP13 fluorescence after IL‐1β stimulation, with partial recovery observed in both the CBZ/COM and CBZ/WCOM groups (Figure 3J,K). The corresponding quantitative analyses are shown in Figure S3.

Similarly, IL‐1β markedly increased the expression of inflammatory mediator‐related genes in chondrocytes. Compared with the IL‐1β group, the COM group exhibited only limited attenuation, whereas both the CBZ/COM and CBZ/WCOM groups reduced the expression of these inflammatory genes (Figure S3A–C). Western blotting further showed that COX2 protein expression was markedly elevated by IL‐1β and was decreased after treatment with either CBZ/COM or CBZ/WCOM, while COM alone showed only a minor effect (Figure S3D,E). Consistently, immunofluorescence staining showed enhanced COX2 signals in the IL‐1β group, which were attenuated in both the CBZ/COM and CBZ/WCOM groups (Figure S3F), with quantitative analysis shown in Figure S3G.

### 2.4 CBZ/WCOM Modulates Nav1.7‐Related Sodium Channel Activity, Na⁺/Ca²⁺ Exchanger (NCX)‐Associated Ca^2^^+^ Regulation, and Downstream Secretory Responses in Chondrocytes

**FIGURE 4:**
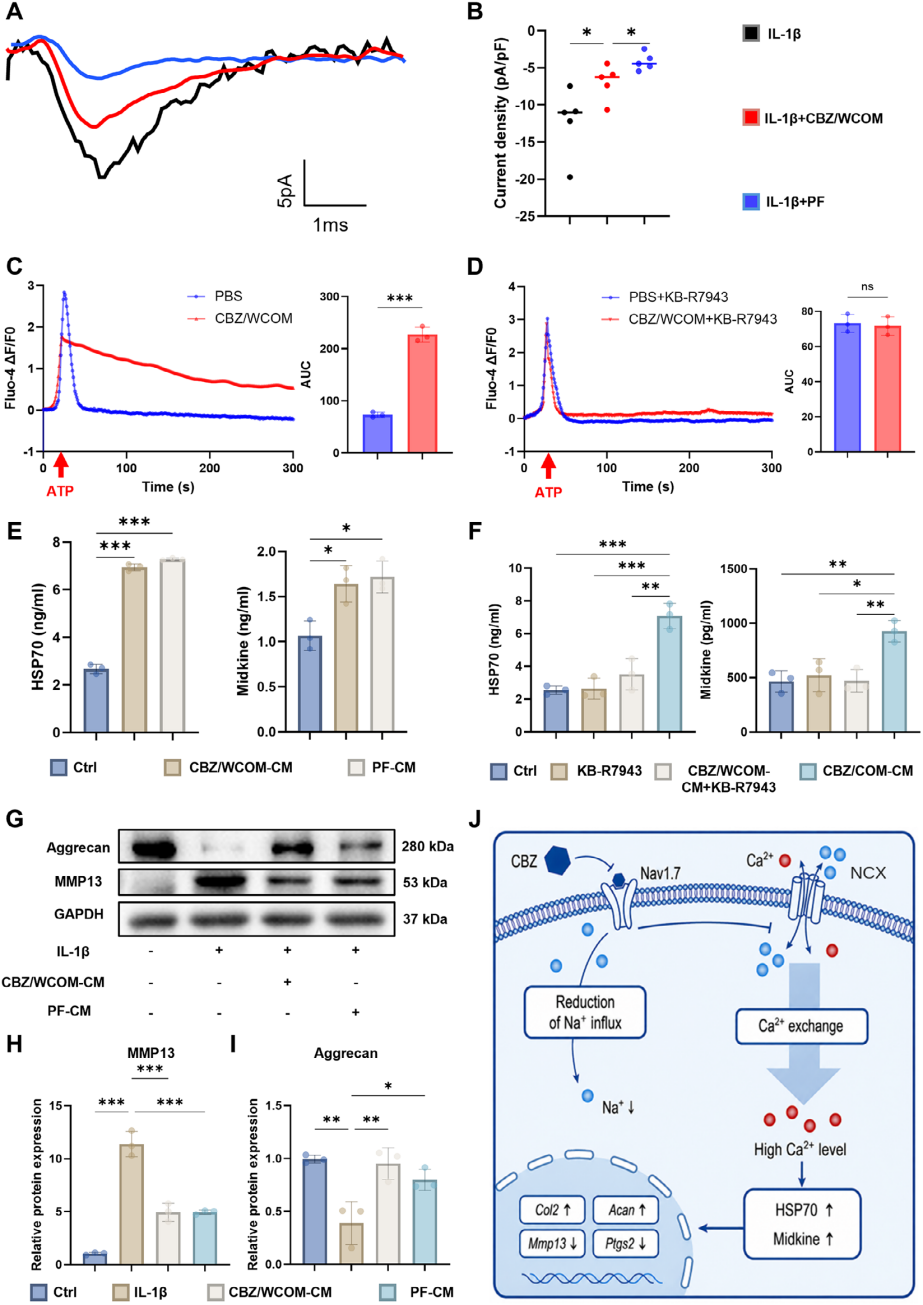
CBZ/WCOM modulates Nav1.7‐related sodium channel activity, NCX‐associated Ca^2^^+^ regulation, and downstream secretory responses in chondrocytes. (A) Superimposed representative whole‐cell sodium current traces recorded from IL‐1β‐stimulated chondrocytes. (B) Quantification of the maximum sodium current density. (C) Averaged ΔF/F0 traces of ATP‐evoked intracellular Ca^2^^+^ responses in PBS‐ and CBZ/WCOM‐treated chondrocytes, together with quantification of the area under the curve (AUC). (D) Averaged ΔF/F0 traces of ATP‐evoked intracellular Ca^2^^+^ responses in PBS + KB‐R7943 and CBZ/WCOM + KB‐R7943 groups, together with quantification of the AUC. (E) ELISA analysis of HSP70 and Midkine levels in culture supernatants collected from Ctrl, CBZ/WCOM‐treated, and PF‐treated chondrocytes. (F) ELISA analysis of HSP70 and Midkine levels in culture supernatants after treatment with the Na^+^/Ca^2^^+^ exchange inhibitor KB‐R7943. (G) Western blot analysis of MMP13 and Aggrecan expression in chondrocytes treated with conditioned media from the indicated groups. (H, I) Quantification of MMP13 and Aggrecan protein expression. (J) Schematic illustration of the proposed mechanism by which CBZ/WCOM regulates Nav1.7‐related sodium channel activity, NCX‐associated Ca^2^^+^ signaling, and downstream HSP70/Midkine secretion in chondrocytes. Data are presented as mean ± SD. For patch‐clamp quantification in (B), *n* = 5. For Ca^2^^+^ imaging analyses in (C, D), *n* = 3 independent experiments. For ELISA and western blot quantification in (E, F, H, I), *n* = 3. ^*^ *p* < 0.05; ^**^ *p* < 0.01; ^***^ *p* < 0.001.

To further explore the mechanism underlying the effect of CBZ, whole‐cell patch‐clamp recording was performed in IL‐1β‐stimulated chondrocytes. As shown in Figure 4A, both CBZ/WCOM and PF suppressed the sodium current induced after IL‐1β stimulation. The inhibitory effect of CBZ/WCOM was slightly weaker than that of PF, but the overall trend was comparable. Quantitative analysis of current density showed that CBZ/WCOM reduced sodium current density compared with the IL‐1β group, whereas PF produced a slightly greater reduction than CBZ/WCOM (Figure 4B). The representative current traces and the corresponding current–voltage relationship are shown in Figure S4A,B. Calcium imaging after adenosine triphosphate (ATP) stimulation further showed that, compared with the PBS group, CBZ/WCOM attenuated the early ATP‐evoked Ca^2^^+^ peak, while maintaining a relatively sustained later‐phase Ca^2^^+^ signal (Figure 4C). Representative fluorescence are shown in Figure S4C. After treatment with the Na^+^/Ca^2^^+^ exchange inhibitor KB‐R7943, this calcium pattern was no longer observed (Figure 4D), and the representative fluorescence images corresponding to Figure 4D are shown in Figure S4D.

Enzyme‐linked immunosorbent assay (ELISA) analysis further showed that both CBZ/WCOM and PF increased the levels of HSP70 and Midkine in the culture supernatant of chondrocytes (Figure 4E), whereas this increase was reduced after KB‐R7943 treatment (Figure 4F). To further evaluate the downstream effects of these secreted factors, conditioned medium collected from chondrocytes treated with CBZ/WCOM or PF was applied to IL‐1β‐stimulated chondrocytes. Western blotting showed that these conditioned media increased Aggrecan expression and reduced MMP13 expression (Figure 4G–I). These findings were summarized schematically in Figure 4J. In addition, neutralization and rescue experiments further supported the involvement of HSP70 and Midkine (Figure S5). Specifically, HSP70 neutralization was performed in Figure S5A, Midkine neutralization in Figure S5B, recombinant HSP70 rescue in Figure S5C, and recombinant Midkine rescue in Figure S5D. These results showed that HSP70 was associated with the promotion of cartilage matrix synthesis, whereas Midkine was associated with the suppression of matrix degradation.

### 2.5 In Vivo Analgesia and Inhibition of Neural Remodeling

**FIGURE 5:**
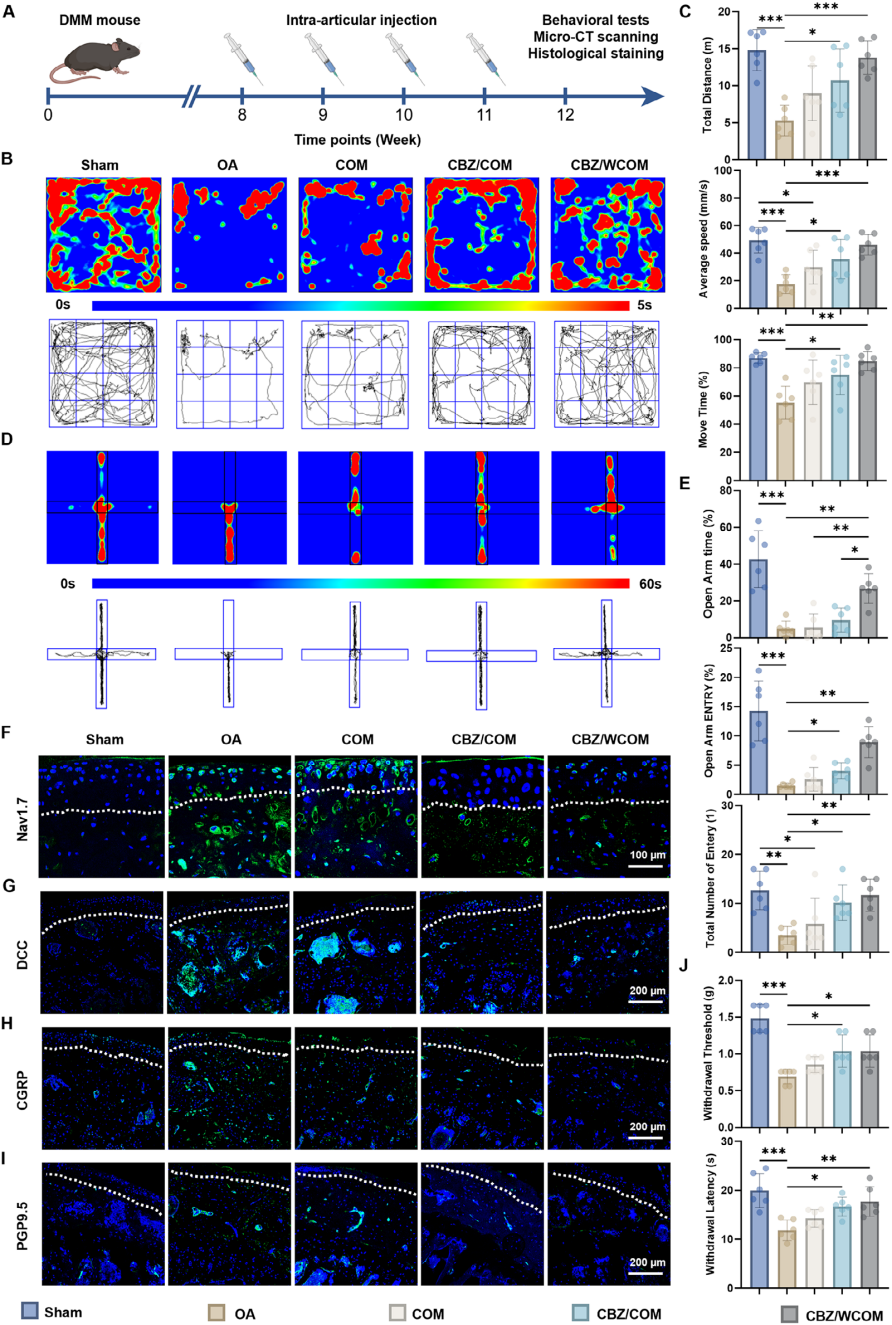
Assessment of pain‐related behaviors and nociceptive innervation after intra‐articular microsphere administration in DMM‐induced osteoarthritis. (A) Experimental timeline of the destabilization of the medial meniscus (DMM) model, intra‐articular injection schedule, and subsequent behavioral, micro‐CT, and histological assessments. (B) Representative open‐field heatmaps (top, 0–5 s color scale) and movement trajectories (bottom) for each experimental group. (C) Quantification of behavioral performance in open‐field assays. (D) Representative elevated‐plus maze heatmaps (top, 0–60 s color scale) and trajectories (bottom). (E) Quantification of behavioral performance in elevated‐plus maze assays. (F) Immunofluorescence staining of Nav1.7 (green) in knee joint sections. Dotted lines indicate the osteochondral interface. Nuclei were counterstained with DAPI (blue). (G–I) Immunofluorescence staining of DCC, CGRP, and PGP9.5 (green). Dotted lines mark the osteochondral boundary. Nuclei were counterstained with DAPI (blue). (J) Evoked pain assays including von Frey mechanical thresholds (top) and hot‐plate withdrawal latencies (bottom) for each group. Data are presented as mean ± SD; *n* = 6. ^*^ *p* < 0.05; ^**^ *p* < 0.01; ^***^ *p* < 0.001.

Moderate‐to‐late OA was established in mice using the destabilization of the medial meniscus (DMM) model, whereas sham‐operated mice underwent arthrotomy without destabilization. Intra‐articular injection was initiated at 8 weeks after surgery and administered once weekly for four consecutive weeks, yielding the following treatment groups: OA, COM, CBZ/COM, CBZ/WCOM, and sham (PBS). Behavioral assessments were performed at week 12 with blinded analysis, together with micro‐CT evaluation and histological assessment (Figure 5A). In the open‐field test, OA and COM mice showed reduced total distance, mean velocity, and effective movement time, whereas both CBZ/COM and CBZ/WCOM improved these parameters relative to the OA and COM groups, showing a similar overall trend of functional recovery (Figure 5B,C). In the elevated‐plus maze, OA and COM mice exhibited reduced open‐arm exploration, while both CBZ/COM and CBZ/WCOM increased open‐arm entries and dwell time compared with the OA and COM groups (Figure 5D,E). Consistently, evoked pain tests showed that both CBZ/COM and CBZ/WCOM increased von Frey thresholds and hot‐plate latencies relative to the OA and COM groups, with broadly comparable analgesic effects under the present experimental conditions (Figure 5J).

Immunofluorescence analysis of knee joint sections showed reduced signals of pain‐related ion channel and neural remodeling markers after intra‐articular CBZ‐loaded microsphere treatment. Nav1.7 immunoreactivity in chondrocytes and the subchondral region was lower in the CBZ/COM and CBZ/WCOM groups than in the OA and COM groups (Figure 5F), with the corresponding quantitative analysis shown in Figure S6A. Similarly, DCC immunoreactivity in the subchondral region was reduced after CBZ/COM and CBZ/WCOM treatment (Figure 5G), with quantification shown in Figure S6B. In addition, the aberrant distribution of CGRP along trabecular bone surfaces observed in the OA and COM groups was attenuated in both the CBZ/COM and CBZ/WCOM groups (Figure 5H), with quantification shown in Figure S6D. PGP9.5 staining also showed reduced nerve‐fiber density after treatment (Figure 5I), with the corresponding quantitative analysis shown in Figure S6C. Collectively, these results showed that intra‐articular administration of CBZ‐loaded microspheres alleviated OA‐associated pain‐related behaviors and reduced pathological neural remodeling in the joint, with CBZ/COM and CBZ/WCOM exhibiting comparable overall effects.

### 2.6 In Vivo Structural Protection in DMM‐Induced Osteoarthritis

**FIGURE 6:**
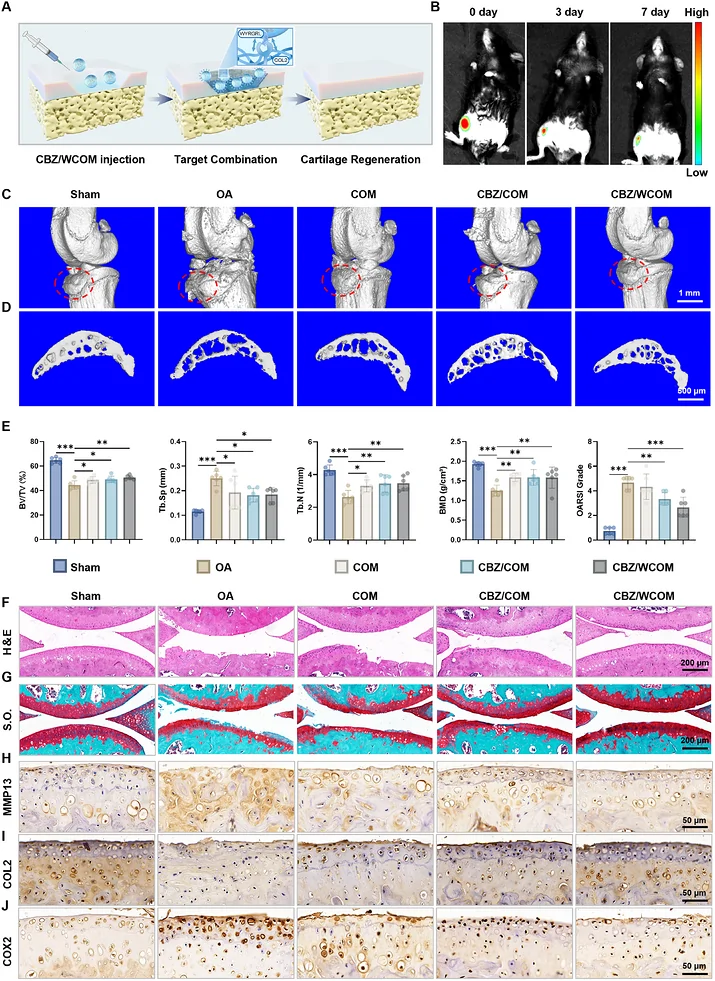
Evaluation of intra‐articular microsphere‐mediated cartilage and subchondral bone protection in DMM‐induced osteoarthritis. (A) Schematic illustration of CBZ/WCOM‐mediated cartilage targeting through WYRGRL–COL2 interaction. (B) In vivo fluorescence images of Cy5‐labeled CBZ/WCOM in the knee joint at 0, 3, and 7 days after intra‐articular injection. (C) Three‐dimensional micro‐CT reconstructions of knee joints from the indicated groups. Red dashed circles indicate the joint regions of interest. (D) The cross‐sectional micro‐CT images of tibial subchondral bone. (E) Quantitative analysis of micro‐CT parameters, including BV/TV, Tb.Th, Tb.N, Tb.Sp, and BMD, together with OARSI histological scores. (F, G) H&E and Safranin O–Fast Green staining images of knee joint sections. (H–J) Immunohistochemical staining images of MMP13, COL2, and COX2 in cartilage sections. Data are presented as mean ± SD; *n* = 6. ^*^ *p* < 0.05; ^**^ *p* < 0.01; ^***^ *p* < 0.001.

After intra‐articular injection, cyanine 5 (Cy5)‐labeled CBZ/WCOM microspheres remained detectable in the joint cavity for several days, with fluorescence signals observed at days 0, 3, and 7 after administration (Figure 6B). At week 12 after DMM surgery, gross observation and micro‐CT reconstruction showed that both CBZ/COM and CBZ/WCOM reduced OA‐associated joint structural damage compared with the OA and COM groups, including reduced osteophyte formation and partial preservation of joint space (Figure 6C,D, and Figure S7A–C). Quantitative analysis of subchondral bone showed corresponding changes in bone volume fraction (BV/TV), trabecular thickness (Tb.Th), trabecular number (Tb.N), trabecular separation (Tb.Sp), and bone mineral density (BMD), together with Osteoarthritis Research Society International (OARSI) histological scores (Figure 6E). Histological staining further showed that cartilage surface irregularity, focal defects, and proteoglycan loss in the OA and COM groups were alleviated in both the CBZ/COM and CBZ/WCOM groups, as shown by hematoxylin and eosin (H&E) and Safranin O–Fast Green staining (Figure 6F,G). Representative immunohistochemical staining showed higher COL2 expression and lower MMP13 and COX2 expression in the CBZ/COM and CBZ/WCOM groups than in the OA and COM groups (Figure 6H–J), and the corresponding quantitative analyses are presented in Figure S7D–F. Overall, CBZ/COM and CBZ/WCOM showed similar trends across these structural and histological assessments, with numerically greater improvement in some readouts in the CBZ/WCOM group.

To further address whether selective Nav1.7 blockade could reproduce the protective trend observed with CBZ/WCOM, we additionally evaluated PF delivered by the WCOM system in a limited in vivo validation experiment. PF/WCOM showed a protective pattern broadly comparable to CBZ/WCOM in the available readouts, supporting the involvement of Nav1.7‐related signaling in the therapeutic response (Figure S8).

## 3 Discussion

Microsphere therapy for OA has recently become a research hotspot [28]. Conventional intra‐articular agents typically show short residence times, pronounced peak–trough fluctuations, and off‐target risk [29], and they inadequately address pain [30]—the defining clinical burden and a driver of disease progression [4, 31]. Herein, we engineered a COL2–targeting, pH‐responsive platform (CBZ/WCOM) that delivers coordinated pharmacokinetic and pharmacodynamic advantages: a WYR peptide homes the carrier to cartilage lesions by recognizing exposed type II collagen, while an acid‐labile Schiff‐base linkage triggers on‐demand drug release to treat OA in the mildly acidic, enzyme‐rich synovial milieu. Together, these features sustain stable, therapeutically relevant perilesional exposure to CBZ to support durable analgesia, while minimizing systemic exposure and attendant adverse effects.

In OA, the superficial zone that normally shields the articular cartilage is progressively eroded by mechanical overload, inflammatory mediators, and enzyme‐driven matrix loss [3, 24]. Early events include depletion of proteoglycans (aggrecan) and cleavage of COL by MMPs/ADAMTS, which disrupt the collagen–proteoglycan meshwork, cause fibrillation, and expose COL2 fibers at the cartilage lesions [21]. As synovitis and osteochondral remodeling intensify, shear and compressive stresses further denude the matrix, expanding COL2–positive, cell‐poor regions [6, 32]. This lesion‐restricted exposure of COL2 creates a stable, extracellular target that persists across disease stages and is directly linked to sites of degeneration. Therefore, we chose COL2 as the target for CBZ/WCOM rather than cell markers or generic physicochemical cues. Compared with receptors, such as CD44 or integrins (expression varies by cell type, inflammation state, and depth) [33, 34], COL2 is abundant, structurally stable, and uniformly accessible wherever the cartilage surface is breached. Targeting COL2 therefore offers (i) spatial precision to degeneration fronts, (ii) stage robustness across heterogeneous lesions, and (iii) low off‐target binding in synovium and subchondral compartments that lack exposed cartilage matrix [19]. By anchoring via a WYR peptide to exposed COL2 and coupling this with an acid‐labile Schiff‐base linkage, CBZ/WCOM achieves “bind‐then‐release” behavior: firm localization at lesions and on‐demand drug liberation in the mildly acidic, protease‐rich joint.

Mechanistically, this study focused on the involvement of Nav1.7‐related sodium channel activity in chondrocyte metabolic regulation. Nav1.7 is known as a key regulator of nociceptor excitability and has also been reported in chondrocytes, where altered Na^+^ influx may affect intracellular Ca^2^^+^ homeostasis and downstream catabolic or inflammatory signaling [8]. Whole‐cell patch‐clamp recordings showed that CBZ/WCOM reduced sodium current density in IL‐1β‐stimulated chondrocytes, and a comparable inhibitory trend was observed with the selective Nav1.7 inhibitor PF. These results provide functional evidence that sodium channel activity was modulated under inflammatory conditions and support the involvement of Nav1.7‐related signaling in CBZ/WCOM‐mediated chondrocyte regulation.

The involvement of NCX‐associated Ca^2^^+^ regulation downstream of Nav1.7‐related sodium channel modulation was further supported by calcium imaging and pharmacological inhibition experiments. Quantitative ΔF/F_0_ and AUC analyses showed that CBZ/WCOM modulated ATP‐evoked intracellular Ca^2^^+^ responses, whereas the Na^+^/Ca^2^^+^ exchange inhibitor KB‐R7943 attenuated this response pattern. ELISA analysis further showed that CBZ/WCOM and PF increased HSP70 and Midkine secretion, while this effect was reduced after KB‐R7943 treatment. Conditioned‐medium transfer, neutralization, and rescue experiments supported the contribution of these secreted factors to matrix regulation, with HSP70 associated with anabolic matrix responses and Midkine associated with suppression of matrix catabolism [8, 10]. Together, these data suggest a functional link among sodium channel modulation, NCX‐associated Ca^2^^+^ regulation, and HSP70/Midkine‐related secretory responses.

In an animal study, CBZ‐loaded microspheres alleviated OA‐associated pain behaviors, reduced pain‐related neural remodeling, and partially preserved cartilage and subchondral bone structure in the DMM model. Both CBZ/COM and CBZ/WCOM improved behavioral and structural readouts compared with OA or COM controls, while CBZ/WCOM showed numerically greater improvement in selected parameters. However, these data do not support the overstatement that CBZ/WCOM is universally superior to CBZ/COM in all biological outcomes. Instead, the main advantage of WYR modification appears to be improved lesion localization and retention, whereas the therapeutic effects are largely driven by sustained local delivery of CBZ.

Because CBZ is a clinically used but non‐selective sodium channel inhibitor, we additionally evaluated PF delivered by the same WCOM platform. Although this experiment included a limited set of groups and was designed as a mechanistic validation rather than a full therapeutic comparison, PF/WCOM showed a protective trend broadly comparable to CBZ/WCOM in the available structural and behavioral readouts. This finding supports the involvement of Nav1.7‐related signaling in the therapeutic response. At the same time, CBZ/WCOM was retained as the main formulation because CBZ is an established clinically used drug, which may facilitate translational development.

Several limitations should be acknowledged. First, CBZ may affect sodium channel subtypes other than Nav1.7; therefore, the therapeutic effects of CBZ/WCOM should be interpreted as Nav1.7‐related rather than exclusively Nav1.7‐specific. Second, because KB‐R7943 is a pharmacological inhibitor of Na^+^/Ca^2^^+^ exchange activity, these experiments support the involvement of NCX‐associated Ca^2^^+^ regulation but do not establish NCX1‐specific causality or exclude other Ca^2^^+^ regulatory pathways. Third, PF/WCOM was performed as a supplementary validation with limited grouping, and more complete in vivo comparisons would further strengthen the specificity claim. Finally, long‐term safety, dosing intervals, repeated‐injection effects, and systemic exposure require further evaluation. Overall, CBZ/WCOM integrates cartilage lesion targeting, pH‐responsive drug release, and local sodium channel modulation to achieve combined analgesic and chondroprotective effects, providing a translationally relevant strategy for local OA therapy.

## 4 Conclusion

In this study, we developed cartilage‐targeted, microenvironment‐responsive hydrogel microspheres (CBZ/WCOM) as an intra‐articular delivery platform for osteoarthritis therapy. By integrating WYRGRL‐mediated recognition of exposed type II collagen with reversible Schiff‐base chemistry, this system enabled lesion‐oriented microsphere retention and acidic microenvironment‐triggered release of carbamazepine. The chondroitin sulfate/oxidized hyaluronan matrix further provided favorable biocompatibility, biodegradability, and chondro‐supportive properties. Through these integrated features, CBZ/WCOM partially restored chondrocyte metabolic balance, attenuated inflammatory and catabolic responses, reduced pain‐related neural remodeling, preserved cartilage matrix integrity, and ameliorated subchondral bone alterations in the DMM‐induced OA model. These findings suggest that CBZ/WCOM may offer a minimally invasive and locally sustained therapeutic strategy with analgesic and structure‐protective potential for osteoarthritis treatment.

## 5 Experimental Section

### 5.1 Preparation and Characterization of CBZ/WCOM Microspheres

To synthesize oxidized hyaluronic acid (OHA), hyaluronic acid (HA, 1.5 g) was first dissolved in 150 mL of deionized water under gentle stirring to obtain a clear solution. Subsequently, sodium periodate (NaIO_4_, 802 mg) was added to oxidize the vicinal diols on the HA backbone, and the reaction was allowed to proceed for 2 h at room temperature in the dark. The oxidation reaction was terminated by adding 200 µL of ethylene glycol to quench the excessive NaIO_4_, followed by dialysis against deionized water for 3 days to remove residual reagents. The purified OHA solution was then lyophilized to obtain the oxidized HA for subsequent use. Composite microspheres (COM) were produced by a microfluidic process. Briefly, methacrylated chondroitin sulfate (ChSMA, EFL, China), OHA, and lithium phenyl‐2,4,6‐trimethylbenzoylphosphinate (LAP, EFL, China) were dissolved in deionized water at a mass ratio of 20: 4: 1 (w/w/w) to form the dispersed phase. The continuous phase consisted of mineral oil (ST1524, Beyotime, China) containing 5% (w/w) Span‐80 (S110839, Aladdin, China). Monodisperse droplets were generated by adjusting the flow‐rate ratio of the continuous to dispersed phases (Qc: Qd = 20: 1). Collected droplets were photocrosslinked under 405 nm UV light for 2 min, washed repeatedly with 75% ethanol and deionized water, and lyophilized to obtain 1% COM microspheres. CM and 0.5% COM were fabricated analogously. CM contained no OHA, and 0.5% COM contained half the OHA used for 1% COM. To obtain CBZ/WCOM, 1% COM microspheres were dispersed in a CBZ solution (15 mg/mL in 75% ethanol) and reacted at 37°C under light protection for 24 h. Microspheres were collected by centrifugation, washed three times with deionized water to remove free CBZ, and lyophilized to yield CBZ/COM. Then CBZ/COM was activated with N‐hydroxysuccinimide (NHS, Aladdin, China) and 1‐(3‐dimethylaminopropyl)‐3‐ethylcarbodiimide hydrochloride (EDC, Aladdin, China) at room temperature with shaking (300 rpm, 30 min). The activated CBZ/COM microspheres were washed three times with PBS and incubated with WYRGRL peptide (MCE, China) in 1 mL PBS at 37°C for 2 h. After centrifugation (3000 g, 10 min), the pellet was resuspended in PBS to obtain CBZ/WCOM.

Lyophilized microspheres were observed by scanning electron microscopy (SEM, Zeiss, Germany). Size and morphology were assessed by bright‐field microscopy (Zeiss, Germany). FTIR (Thermo Fisher, USA) was used to verify peptide conjugation and CBZ linkage. All measurements were performed in triplicate.

To obtain PF/WCOM microspheres, 1% COM microspheres were dispersed in PF‐04856264 solution (1 mg/mL in 75% ethanol; MCE, China) and incubated at 37°C under light protection for 24 h. The microspheres were then collected by centrifugation, washed three times with PBS to remove unloaded PF‐04856264, and lyophilized to yield PF/COM microspheres. Subsequently, PF/COM microspheres were activated with N‐hydroxysuccinimide (NHS, Aladdin, China), and 1‐(3‐dimethylaminopropyl)‐3‐ethylcarbodiimide hydrochloride (EDC, Aladdin, China) at room temperature with shaking (300 rpm, 30 min). After washing three times with PBS to remove residual EDC/NHS, the activated PF/COM microspheres were incubated with WYRGRL peptide (MCE, China) in 1 mL PBS at 37°C for 2 h. After centrifugation at 3000 × g for 10 min, the pellet was resuspended in PBS to obtain PF/WCOM microspheres. PF/WCOM was prepared using the same carrier, peptide‐conjugation procedure, and final microsphere concentration as CBZ/WCOM and was used as a supplementary mechanistic validation group.

### 5.2 Fluorescent Labeling of WYR Peptide for CBZ/WCOM Targeting Assessment

Targeting peptides were labeled with fluorescein isothiocyanate (FITC, MCE, China). FITC (0.3 mg) was dissolved in 30 µL N, N‐dimethylformamide (DMF) and mixed with the peptide in labeling buffer, followed by incubation at 37°C protected from light for 30 min. FITC‐labeled peptide‐conjugated microspheres were collected by centrifugation and washed repeatedly with PBS until the supernatant became clear. Cy5 labeling (MCE, China) followed an analogous procedure.

### 5.3 Degradation of CBZ/WCOM

Enzymatic degradation of CBZ/WCOM was evaluated in PBS containing type II collagenase (2 µg/mL) and hyaluronidase (2 µg/mL) to simulate physiological enzyme activity. CBZ/WCOM was incubated at 37°C on an orbital shaker (90 rpm), with medium replaced every 2 days. Microsphere morphology and size were recorded by inverted microscopy on days 2, 3, 5, 7, 18, and 24. Samples were then lyophilized to obtain residual dry mass. The dispersion/measurement cycle was repeated to further emulate biodegradation.

### 5.4 Measurement of CBZ Release Behavior

CBZ/WCOM was immersed in 2 mL PBS at pH 5.5 or 7.4 and incubated in a thermoshaker at 37°C (80 rpm). At predetermined intervals (2, 4, 12 h, 1, 3, 5, 7, 14, and 21 d), 1 mL supernatant was collected and replenished with an equal volume of fresh PBS. CBZ concentration was determined by UV–vis spectrophotometry (Agilent, USA) to construct release profiles.

### 5.5 Cell Culture

Primary mouse chondrocytes (mPCs) were isolated from femoral condyles and tibial plateaus of postnatal day‐6 C57 mice according to the previous study [35]. Trimmed cartilage explants were digested in 0.25% trypsin for 10 min at 37°C and then incubated in 0.1% type II collagenase overnight. Cell suspensions were filtered through 100 µm nylon meshes and then cultured in dishes at a cell density of 2.5 × 10^4^ cells/cm^2^. First‐ and second‐passage mPCs were used in experiments. Cells were cultured in complete medium (DMEM/F‐12, Gibco, USA) supplemented with 10% FBS (Gibco, USA) and 1% penicillin–streptomycin (Gibco, USA).

### 5.6 Live/Dead Staining

mPCs were detached with 0.25% trypsin and seeded in 24‐well plates at 1 × 10^5^ cells/well. Each group received 540 µL DMEM/F‐12 with 10% FBS plus 60 µL of CM, 0.5% COM, or 1% COM. After 1, 3, and 5 days, cells were washed with PBS and stained via a Viability/Cytotoxicity kit (Calcein‐AM/EthD‐I, Proteintech, China) for 15 min in the dark. Confocal imaging was performed at 490/525 nm (live) and 525/620 nm (dead) to observe the cell viability.

### 5.7 CCK‐8 Assay

mPCs were seeded in 96‐well plates at 1 × 10^3^ cells/well. For CBZ cytocompatibility evaluation, cells were treated with complete medium containing different concentrations of free CBZ for 24 h, and the optimal concentration was selected for subsequent experiments. For microsphere cytocompatibility evaluation, each treatment well received 90 µL complete medium and 10 µL of CM, 0.5% COM, or 1% COM suspension, while control wells received 100 µL complete medium. At the indicated time points, 10 µL CCK‐8 reagent (NCM Biotech, China) was added to each well and incubated for 2 h at 37°C in 5% CO_2_. The absorbance was then measured at 450 nm.

### 5.8 Ca^2^^+^ Fluorescence Imaging

Primary mouse chondrocytes (mPCs) were seeded in confocal dishes and incubated with Fluo‐4 AM (5 µm; Beyotime, China) in Hanks’ balanced salt solution (HBSS) for 45 min at 37°C in the dark to allow intracellular dye loading. After washing twice with HBSS to remove excess probe, the cells were equilibrated for 10 min before imaging. Fluorescence signals were recorded using a Zeiss LSM 900 confocal microscope equipped with a 20× objective lens, with excitation at 488 nm and emission collected at 500–550 nm. To induce Ca^2^^+^ transients, ATP (5 µm) was added after a stable baseline fluorescence signal had been recorded, and time‐lapse images were acquired for 300 s at 1 s intervals.

For quantitative analysis, regions of interest (ROIs) were drawn around individual cells, and the mean fluorescence intensity of each ROI was measured at each time point. Ca^2^^+^ responses were expressed as ΔF/F0, which was calculated as (F–F0)/F0, where F represents the fluorescence intensity at each time point and F0 was defined as the mean fluorescence intensity during a stable baseline window immediately before the first large ATP‐evoked Ca^2^^+^ rise. The same baseline‐window criterion was applied to all ROIs and groups. The ΔF/F0 curves were plotted over time for each group. The area under the curve (AUC) was then calculated from the ΔF/F0 traces over the recording period to quantify the overall Ca^2^^+^ response.

For Na^+^/Ca^2^^+^ exchange inhibition experiments, cells were pretreated with KB‐R7943 mesylate (10 µm; MCE, China) for 30 min before ATP stimulation, and KB‐R7943 was maintained in HBSS throughout the imaging period. Vehicle‐treated cells received the same final concentration of DMSO. Ca^2^^+^ imaging and ΔF/F0/AUC analyses were performed as described above.

### 5.9 Quantitative Real‐Time PCR (RT‐qPCR)

mPCs were seeded in 6‐well plates at 2.5 × 10^4^ cells/cm^2^. For the free‐drug validation experiments shown in Figure 1, cells were stimulated with IL‐1β (10 ng/mL) and treated with free CBZ (10 µm) or PF‐04856264 (1 µm) for 24 h. Negative control cells received complete medium without IL‐1β, and positive control cells received IL‐1β only. For the microsphere treatment experiments shown in Figure 3 and Figure S3, treatment wells received 3 mL DMEM/F‐12 containing 10% FBS and IL‐1β (10 ng/mL) plus COM, CBZ/COM, or CBZ/WCOM. After 24 h of treatment, total RNA was extracted using TRIzol reagent, and cDNA was synthesized using HiFiScript (CWBiO, China). The primers were synthesized by Sangon Biotech (Shanghai, China), and the primer sequences used for RT‐qPCR are listed in Table 1.

**TABLE 1:** PCR primer sequences.

Gene	Forward Primer (5′→3′)	Reverse Primer (5′→3′)
*18S*	CGGCTACCACATCCAAGGAA	GCTGGAATTACCGCGGCT
*Col2a1*	GGTGGAGCAGCAAGAGCAAGG	CCAGGTTGCCATCGCCATAGC
*Mmp13*	GTCCCTGCCCCTTCCCTATGG	CGCAAGAGTCGCAGGATGGTAG
*Scn9a*	AAGATGGAGACAGAGATGACGATT	GGAAGGTGGAGAGATGGTAGAG
*Il6*	TTCTTGGGACTGATGCTGGTGAC	CTGTTGGGAGTGGTATCCTCTGTG
*Ptgs2*	TGAGTACCGCAAACGCTTCT	CAGCCATTTCCTTCTCTCCTGT
*Adamts5*	CCCAGGATAAAACCAGGCAG	CGGCCAAGGGTTGTAAATGG
*Acan*	GTGGAGCCGTGTTTCCAAG	AGATGCTGTTGACTCGAACCT
*Nos2*	ACTCAGCCAAGCCCTCACCTAC	CGTCTCGTCCGTGGCAAAGC
*Mmp3*	TCGGGTTGGAGATGACAGGGAAG	TGAAGCCACCAACATCAGGAACAC

qPCR was performed with 2× NovoStart SYBR qPCR SuperMix (Novoprotein, China) on a QuantStudio 7 Real‐Time PCR System (Thermo Fisher Scientific, USA). Relative gene expression was calculated using the 2^−ΔΔCt^ method with 18S as the internal reference gene.

### 5.10 Immunofluorescence Staining

mPCs were seeded in 24‐well plates at 1 × 10^5^ cells/well. For the free‐drug validation experiments shown in Figure 1, cells were stimulated with IL‐1β (10 ng/mL) and treated with free CBZ (10 µm) or PF‐04856264 (PF, 1 µm) for 24 h. Negative control cells received complete medium without IL‐1β, and positive control cells received IL‐1β only. For the microsphere treatment experiments shown in Figure 3 and Figure S3, treatment wells received 540 µL DMEM/F‐12 containing 10% FBS and IL‐1β (10 ng/mL) plus 60 µL of COM, CBZ/COM, or CBZ/WCOM suspension. After 24 h of treatment, cells were washed three times with PBS and fixed with 4% paraformaldehyde for 15 min. After additional PBS washes, coverslips or microsphere‐associated cell samples were blocked with commercial blocking buffer (Beyotime, China) for 1 h and incubated with primary antibodies overnight at 4°C, including rabbit anti‐Nav1.7, rabbit anti‐COL2 (Proteintech, China), rabbit anti‐MMP13 (Thermo Fisher, USA), and rabbit anti‐COX2 (Abcam, USA). After washing with PBS, samples were incubated with the appropriate fluorescent secondary antibodies and/or Actin‐Tracker Red‐Rhodamine (1:500) for 30 min at room temperature in the dark. Samples were then washed with PBS and counterstained with DAPI for 10 min. Fluorescence images were acquired using confocal microscopy under consistent imaging settings.

### 5.11 Western Blotting

For the free‐drug validation experiments shown in Figure 1, mPCs were stimulated with IL‐1β (10 ng/mL) and treated with free CBZ (10 µm) or PF‐04856264 (PF, 1 µm) for 24 h. For microsphere‐related experiments, cells were treated with IL‐1β (10 ng/mL) together with COM, CBZ/COM, or CBZ/WCOM according to the experimental design. After treatment, cells were washed with PBS and lysed on ice for 30 min in RIPA buffer containing protease and phosphatase inhibitors (Roche, Switzerland). Protein concentration was determined, and equal amounts of protein were separated by SDS‐PAGE and transferred to PVDF membranes. Membranes were blocked with milk, incubated with primary antibodies overnight at 4°C, washed with TBST, and incubated with HRP‐conjugated secondary antibodies for 60 min at room temperature. Protein bands were visualized by ECL and quantified using ImageJ.

### 5.12 ELISA

After 48 h of the indicated treatments, culture supernatants were collected and centrifuged at 1000 × g for 10 min at 4°C to remove cell debris. For NCX inhibition experiments, chondrocytes were pretreated with KB‐R7943 mesylate (10 µm; MCE, China) for 30 min before subsequent treatment, and the inhibitor was maintained during the incubation period. The concentrations of Midkine and HSP70 in the culture supernatants were measured using a Mouse Midkine/MDK ELISA Kit (BOSTER, China) and a Mouse HSP70 ELISA Kit (Elabscience, China), respectively, according to the manufacturers’ instructions. Absorbance was measured at 450 nm using a microplate reader, and the concentrations were calculated from the corresponding standard curves.

### 5.13 Whole‐Cell Patch‐Clamp Recording

Before electrophysiological recording, chondrocytes were maintained in standard growth medium for 48 h. Whole‐cell patch‐clamp recordings were performed at room temperature (22°C–25°C). Sodium currents were recorded using a MultiClamp 700B amplifier and a Digidata 1440A digitizer (Axon Instruments, USA), and data acquisition was controlled using pClamp software. Patch pipettes were pulled from borosilicate glass capillaries using a P‐97 micropipette puller and had a resistance of 2–3.5 MΩ when filled with the intracellular solution.

The intracellular pipette solution contained the following components (in mM): 140 CsF, 10 NaCl, 10 HEPES, 1 EGTA, and 20 glucose. The pH was adjusted to 7.3 with CsOH, and the osmolarity was adjusted to 328 mOsm/L with sucrose. The extracellular bath solution contained the following components (in mM): 145 NaCl, 4 KCl, 2 CaCl_2_, 2 MgCl_2_, 10 HEPES, 10 TEA‐Cl, and 10 glucose. The pH was adjusted to 7.4 with NaOH, and the osmolarity was adjusted to 327 mOsm/L. To evoke inward sodium currents, cells were depolarized from −60 to +50 mV in 10 mV increments, with each voltage step lasting 50 ms. The recorded currents were analyzed using Clampfit software. Current density was calculated by normalizing the peak current amplitude to the membrane capacitance.

### 5.14 Neutralization and Rescue Assays

Conditioned media were collected from CBZ/WCOM‐ or PF‐treated chondrocytes after 48 h of treatment and centrifuged at 1000 × g for 10 min at 4°C to remove cell debris. For antibody neutralization assays, conditioned media were preincubated with anti‐HSP70 neutralizing antibody (2 µg/mL; Thermo Fisher Scientific, MA3‐006, USA), anti‐Midkine neutralizing antibody (2 µg/mL; Thermo Fisher Scientific, 500‐P171 USA), or species‐matched control IgG (2 µg/mL; Thermo Fisher Scientific, USA) for 1 h at 37°C. The antibody‐treated conditioned media were then applied to IL‐1β‐stimulated chondrocytes for 24 h. For rescue experiments, recombinant mouse HSP70 protein (Abcam, ab113187, UK) or recombinant mouse Midkine protein (Thermo Fisher Scientific, Cat. No. 315–25, USA) was added to IL‐1β‐stimulated chondrocytes at the indicated concentrations for 24 h. After treatment, cells were collected for western blot analysis of Aggrecan and MMP13 expression.

### 5.15 Animal Experiments

All animal procedures were approved by the Ethics Committee of Soochow University (protocol number: SUDA20260513A03) and performed in accordance with IACUC guidelines. Male C57BL/6 mice (8 weeks old, 20–25 g; GemPharmatech Co., Ltd., Nanjing, China) were used in this study. For the main therapeutic experiment, 30 mice were used, including 24 DMM‐operated mice and 6 sham‐operated mice. For the supplementary PF/WCOM validation experiment, an additional 18 DMM‐operated mice were used. Thus, a total of 48 mice were included.

DMM surgery was performed under pentobarbital anesthesia as previously described with minor modifications [36]. After confirmation of adequate anesthesia by loss of pedal reflex, the knee was disinfected with povidone‐iodine and 75% ethanol. A 5–7 mm medial parapatellar incision was made, and the joint capsule was opened to expose the medial meniscus. The medial meniscotibial ligament was transected with microscissors to establish the DMM model. The joint cavity was irrigated with sterile saline, and the capsule and skin were closed with 5–0 absorbable sutures. Sham‐operated mice underwent capsulotomy and closure without ligament transection. Animals were allowed to recover on a warming pad and monitored after surgery.

For the main therapeutic experiment, at 8 weeks post‐DMM surgery, 24 DMM mice were randomly assigned to four groups: OA, COM, CBZ/COM, and CBZ/WCOM groups (*n* = 6 per group). Six sham‐operated mice served as surgical controls. Mice received intra‐articular injections once weekly for 4 consecutive weeks. Each knee received 10 µL of PBS or microsphere suspension. COM, CBZ/COM, and CBZ/WCOM were administered at the same microsphere concentration of 10 mg/mL, corresponding to 100 µg microspheres per injection.

For the supplementary PF/WCOM validation experiment, an additional 18 DMM mice were randomly assigned to three groups: OA, PF/WCOM, and CBZ/WCOM groups (*n* = 6 per group). Intra‐articular injections were initiated at 8 weeks after DMM surgery and performed once weekly for 4 consecutive weeks using the same injection volume, microsphere concentration, and treatment schedule as the main therapeutic experiment. Each mouse received 10 µL of PBS, PF/WCOM, or CBZ/WCOM suspension into the knee joint. PF/WCOM and CBZ/WCOM were administered at the same microsphere concentration of 10 mg/mL, corresponding to 100 µg microspheres per injection. This supplementary experiment was designed to evaluate whether selective Nav1.7 inhibition delivered by the same WCOM platform could produce a protective trend comparable to CBZ/WCOM.

At week 12 after DMM surgery, behavioral tests, including open‐field, elevated‐plus maze, von Frey, and hot‐plate assays, were performed in a blinded manner. Mice were then euthanized, and knee joints were collected for micro‐CT reconstruction and histological evaluation, including H&E staining, Safranin O–Fast Green staining, and OARSI scoring.

### 5.16 In Vivo Imaging

Cy5‐labeled CBZ/WCOM (Cy5–CBZ/WCOM) was used to minimize interference from skin autofluorescence. A subset of DMM mice from the main therapeutic experiment was used for in vivo fluorescence imaging (*n* = 6). At 8 weeks after DMM surgery, mice received intra‐articular injections of 10 µL Cy5–CBZ/WCOM into the knee joint. Fluorescence images were acquired at 0, 3, and 7 days post‐injection using an IVIS Spectrum system (PerkinElmer, USA).

### 5.17 Behavioral Testing

OA‐related pain behaviors were assessed starting 8 weeks after DMM and following 4 weeks of treatment, using the elevated plus maze (EPM), open‐field test, von Frey assay, and hot‐plate test. All tests were blinded and conducted between 12:00 and 17:00.

#### 5.17.1 Open Field

Mice were individually placed in a transparent 45 × 45 cm arena under normal lighting and allowed to explore for 5 min.

#### 5.17.2 EPM

A plus‐shaped maze elevated 60 cm above the floor (central platform with two closed and two open arms) was used; mice were recorded for 5 min, and open‐arm entries/time and total entries were analyzed.

#### 5.17.3 Von Frey

Mice were placed on an elevated mesh platform and ascending forces were applied to the plantar surface with filaments (Stoelting). A brisk paw withdrawal was scored as positive. Ten trials per force with 30 s intervals were conducted. The tactile threshold was defined as the force producing five positive responses in ten trials.

#### 5.17.4 Hot Plate

The plate was set to 52°C (cut‐off 45 s). After ≥30 min acclimation, mice were placed on the plate and latency to the first nocifensive response (hind‐paw lick/shake or jump) was recorded. If no response by the cut‐off, animals were removed and assigned the maximum latency. Each mouse was tested once per time point (repeat interval ≥10–15 min). The surface was cleaned with alcohol between animals.

### 5.18 OARSI Scoring

Osteoarthritis severity was evaluated according to the Osteoarthritis Research Society International (OARSI) histopathological scoring system for mouse knee OA [37]. The OARSI grading scale comprises eight levels (0–6) that describe progressive structural cartilage damage. Specifically, grade 0 indicates normal cartilage with an intact surface and uniform Safranin O staining. Grade 0.5 corresponds to a loss or reduction of Safranin O staining without apparent structural alteration. Grade 1 denotes minor fibrillation or surface irregularity. Grade 2 reflects vertical fissures confined to the superficial layer. Grade 3 represents vertical damage involving ≤25% of the cartilage surface. Grade 4 indicates vertical damage affecting 25%–50% of the cartilage surface. Grade 5 corresponds to lesions involving 50%–75% of the surface and grade 6 signifies severe vertical erosion extending over >75% of the cartilage surface with marked matrix loss. Higher scores indicate more advanced osteoarthritic degeneration.

### 5.19 Histological Evaluation

Mice were euthanized by cervical dislocation under anesthesia. Knees were fixed in 4% paraformaldehyde for 48 h and transferred to 75% ethanol. Samples were scanned by micro‐CT. After analysis, joints were processed for paraffin histology. Specimens were decalcified in a rapid decalcifier for 2 days, dehydrated through graded ethanol, cleared with xylene, and embedded in paraffin. Sagittal sections (6 µm) were stained with Safranin O–Fast Green, H&E, immunofluorescence, and immunohistochemistry.

#### 5.19.1 Micro‐CT Acquisition and Analysis

Mouse knee joints were dissected free of soft tissues, fixed 48 h in 4% paraformaldehyde, and scanned using a high‐resolution micro‐CT system (SkyScan 1172, Bruker micro‐CT). Image reconstruction and analysis were performed with NRecon v1.6 and CTAn v1.9 software, respectively. Three‐dimensional visualization was performed using CTVol v2.0 (Bruker micro‐CT). The scanner was set at 50 kVp, 200 µA, and a resolution of 5.7 µm per pixel. Sagittal images of the tibial subchondral bone were obtained for three‐dimensional histomorphometric analysis. The region of interest (ROI) encompassed the entire knee joint and the whole medial compartment of the subchondral bone. Eight consecutive slices from the medial tibial plateau were selected for three‐dimensional reconstruction and quantitative evaluation. The structural parameters analyzed included BV/TV (bone volume fraction), Tb.Th (trabecular thickness), Tb.N (trabecular number), Tb.Sp (trabecular separation), and BMD (bone mineral density).

#### 5.19.2 Immunohistochemistry

Sections were deparaffinized in xylene and rehydrated through 100%, 95%, 80%, and 70% ethanol to water. Antigen retrieval was performed with trypsin (Gibco, USA) for 15 min. Endogenous peroxidase was quenched with 3% H_2_O_2_ for 10 min. After PBS washes (3 × 5 min), sections were blocked for 30 min and incubated with primary antibodies (COL2, MMP13, and COX2) overnight at 4°C. The next day, sections were washed and incubated with HRP‐polymer secondary antibodies for 20–30 min, developed with DAB (30 s–3 min under microscopic control), counterstained with hematoxylin, blued, dehydrated, cleared, and mounted in neutral resin. Immunofluorescence followed similar steps with fluorescent detection.

#### 5.19.3 Safranin O–Fast Green

After deparaffinization/rehydration, sections were stained with 0.2% Safranin O (3–5 min), rinsed, stained with 2% Fast Green (25 s), briefly rinsed with 1% acetic acid, washed, and differentiated in absolute ethanol (≈10 s).

#### 5.19.4 H&E

Standard deparaffinization/rehydration, hematoxylin (5 min), water wash (10 min), brief differentiation, eosin (30 s), and graded ethanol dehydration preceded imaging.

### 5.20 Statistical Analysis

All quantitative data are presented as mean ± SD. Statistical analyses were performed using GraphPad Prism (v10.4.0) and Origin 2024. For comparisons between two groups, the unpaired two‐tailed Student's *t*‐test was used. For comparisons among multiple groups, one‐way analysis of variance (ANOVA) followed by Tukey's multiple‐comparison test was performed. A value of *p* < 0.05 was considered statistically significant. Statistical significance was indicated as * *p* < 0.05; ** *p* < 0.01; *** *p* < 0.001.

## Funding

National Natural Science Foundation of China (32471410, 82402800, 32501170), National Key R&D Program of China (2023YFB3810200, 2023YFB3810201), the Project of Biomedical Basic Research Center (BBRC) of Jiangsu, Soochow University (No. BK20255001), Science and Technology Development Project of Suzhou (SGC202379, SZS2023043), Natural Science Foundation of Jiangsu Province (BK20240797, BK20250804), Jiangsu Basic Research Program (Natural Science Foundation) (BK20240020), Major Special Projects of Science and Technology Plan of Xinjiang Uygur Autonomous Region (2022A03011), Postdoctoral Fellowship Program of CPSF (BX20230253, GZB20230505, 2024M752326), the China Postdoctoral Science Foundation under Grant Number 2023TQ0235, Priority Academic Program Development (PAPD) of Jiangsu Higher Education Institutions, and the Top Talent Support Program for young and middle‐aged people of Wuxi Health Committee (BJ2023106).

## Conflicts of Interest

The authors declare no conflicts of interest.

## Supporting information




**Supporting File**: advs75986‐sup‐0001‐SuppMat.docx.

## Data Availability

The data that support the findings of this study are available from the corresponding author upon reasonable request.
