# Supplementary material for: Lesion Site‐Targeted Microspheres Modulate Nav1.7‐Related Signaling for Osteoarthritis Treatment
Source: Adv Sci (Weinh). 2026 Jun 4:e75986. Online ahead of print. doi: 10.1002/advs.75986 (PMC13336385; doi:10.1002/advs.75986)
Supplement: Supplementary file 1 — Supporting File: advs75986‐sup‐0001‐SuppMat.docx. [file ADVS-9999-e75986-s001.docx]

Supporting Information

**Lesion site-targeted microspheres modulate Nav1.7-related signaling for osteoarthritis treatment**

*Cheng Chen^#1,2,3^, Jiaying Li^#2^, Jinjin Ma^#2^, Xiaonan Yuan^2^, Yao Xiao^2^, Yang Zhang^2^ , Yuan Chen^2^, Hao Jiang^2^, Hui He^2^, Jie Hu^2^ , Qianglong Chen^2^, Jinniu Zhang^4^, Bin Li^2^, Yun Zhou*^4^, Fengxuan Han*^2^, Yong Wang*^1,3^*

^1^ Department of Orthopedic Surgery, the Affiliated Yixing Hospital of Jiangsu University, Wuxi, Jiangsu 214200, China.

^2^ Medical 3D Printing Center, Orthopedic Institute, Department of Orthopedic Surgery, The First Affiliated Hospital, School of Basic Medical Sciences, Interdisciplinary Innovation Center for Nanomedicine, MOE Key Laboratory of Geriatric Diseases and Immunology, Changzhou Geriatric hospital, Suzhou Medical College, Biomedical Basic Research Center of Jiangsu, Soochow University, Suzhou, Jiangsu 215000, China.

^3^ Regenerative Medicine & Tissue Engineering Research Center, Institute of Medical Innovation and Translation, the Affiliated Yixing Hospital of Jiangsu University, Yixing, 214200, China.

^4^ Department of Rehabilitation Medicine, The Second Affiliated Hospital of Anhui Medical University, Hefei, Anhui 230601, China.

C. Chen, J. Li and J. Ma contributed equally to this study.

***Corresponding author. E-mail: zhoukeg@ahmu.edu.cn, fxhan@suda.edu.cn, orthopedist1982@163.com.


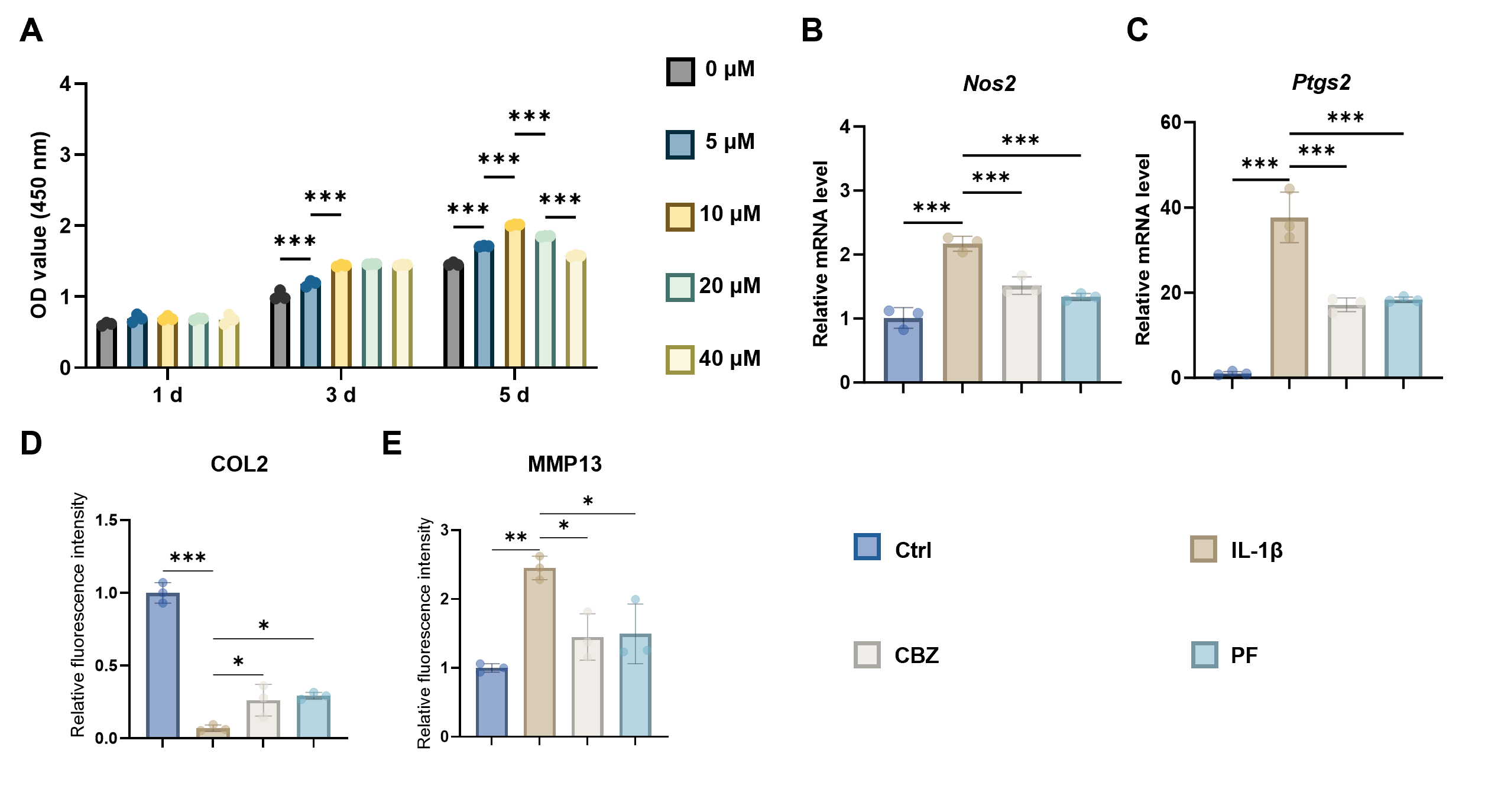


**Figure S1.** Evaluation of CBZ concentration and inflammatory gene expression in chondrocytes. (A) Cell viability determination of chondrocytes by CCK-8 assay after treatment with different concentrations of CBZ. (B, C) RT-qPCR analysis of Nos2 and Ptgs2 mRNA levels in the indicated groups. (D, E) Quantification of COL2 and MMP13 immunofluorescence intensity in the indicated groups. Data are presented as mean ± SD; n = 3. *, *p* < 0.05, **, *p* < 0.01, ***, *p* < 0.001.

**
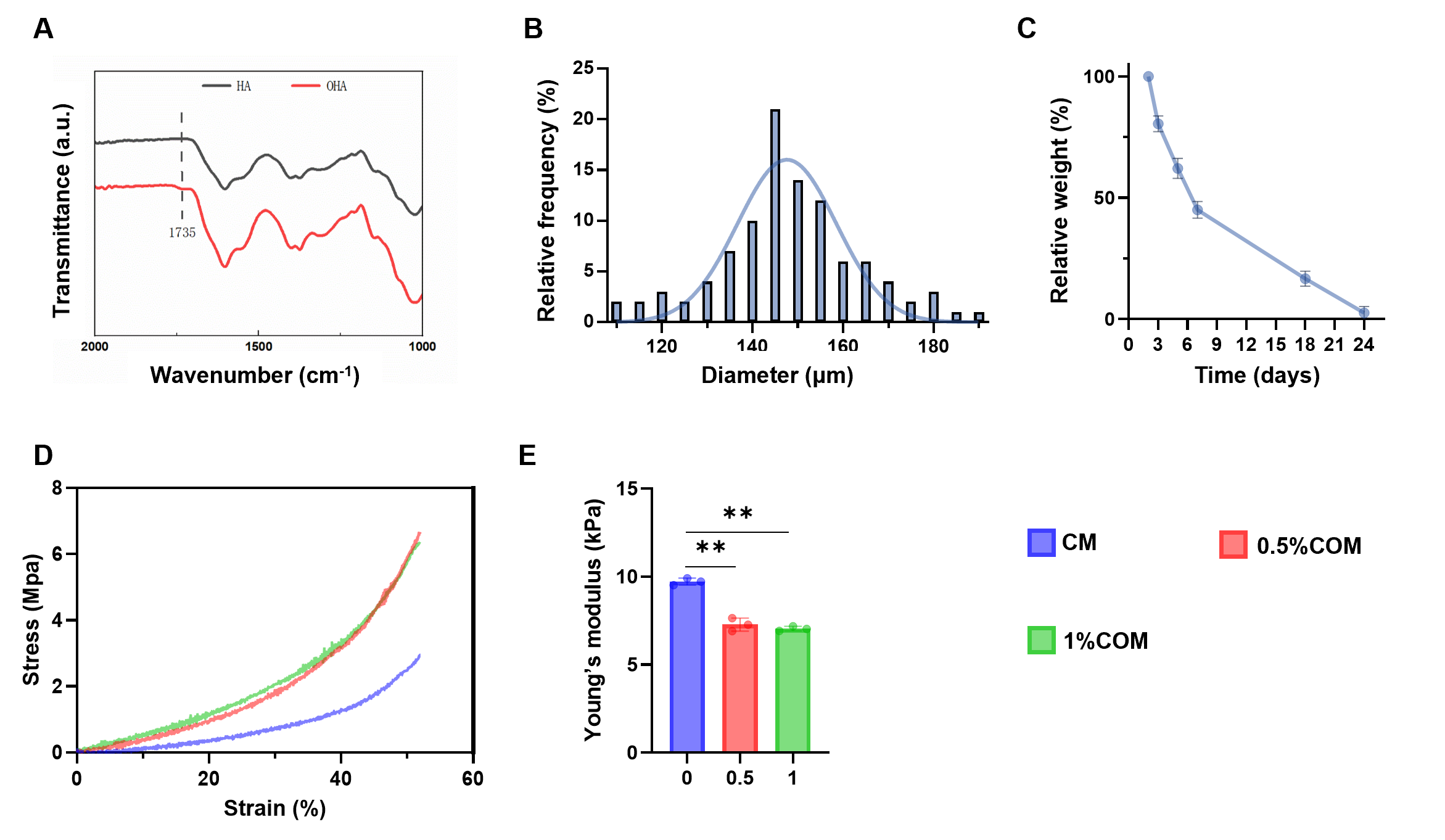
**

**Figure S2.** Physicochemical characterization of microspheres. (A) FTIR spectra of HA and OHA. (B) Diameter distribution of COM. (C) In vitro enzymatic degradation of COM microspheres. (D) Representative stress–strain curves of hydrogels prepared from methacrylated chondroitin sulfate (ChSMA) alone or blended with OHA at 0.5% or 1%. (E) Young’s modulus values calculated from the initial linear region of the curves shown in (D). Data are presented as mean ± SD; n = 3. **, *p* < 0.01.


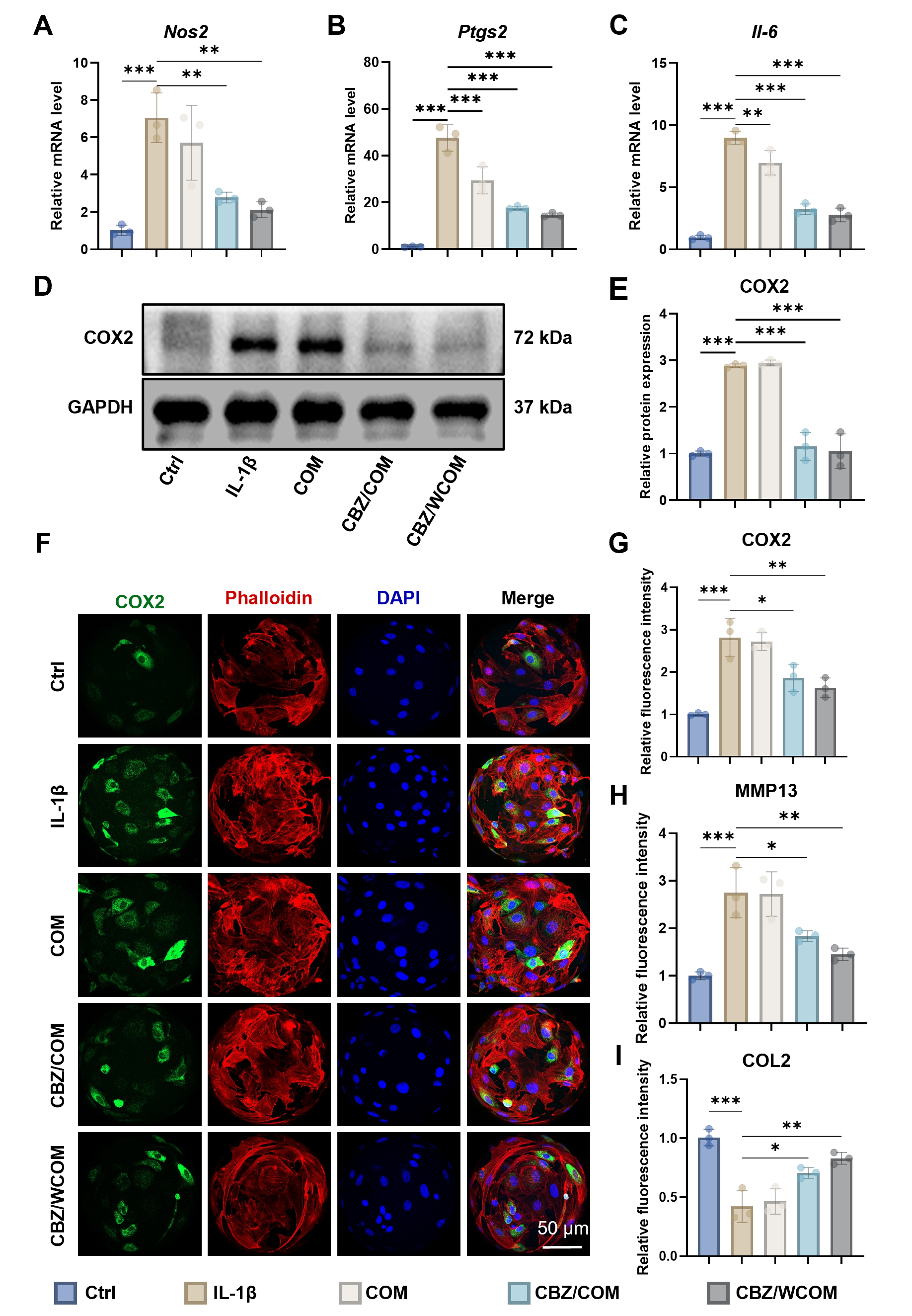


**Figure S3.** Evaluation of the anti-inflammatory effects of microspheres in chondrocytes under inflammatory conditions. (A–C) RT-qPCR analysis of the mRNA levels of inflammatory-related genes, including Nos2, Ptgs2, and Il6, in the indicated groups. (D) Western blot analysis of COX2 expression in the indicated groups. (E) Quantification of COX2 protein expression from (D) by densitometric analysis. (F) Immunofluorescence staining of COX2 (green) co-labeled with F-actin (phalloidin, red) and nuclei (DAPI, blue) in chondrocyte spheroids. (G–I) Quantification of COX2, MMP13, and COL2 fluorescence intensity in the indicated groups. Data are presented as mean ± SD; n = 3. *, *p* < 0.05, **, *p* < 0.01, ***, *p* < 0.001.

**
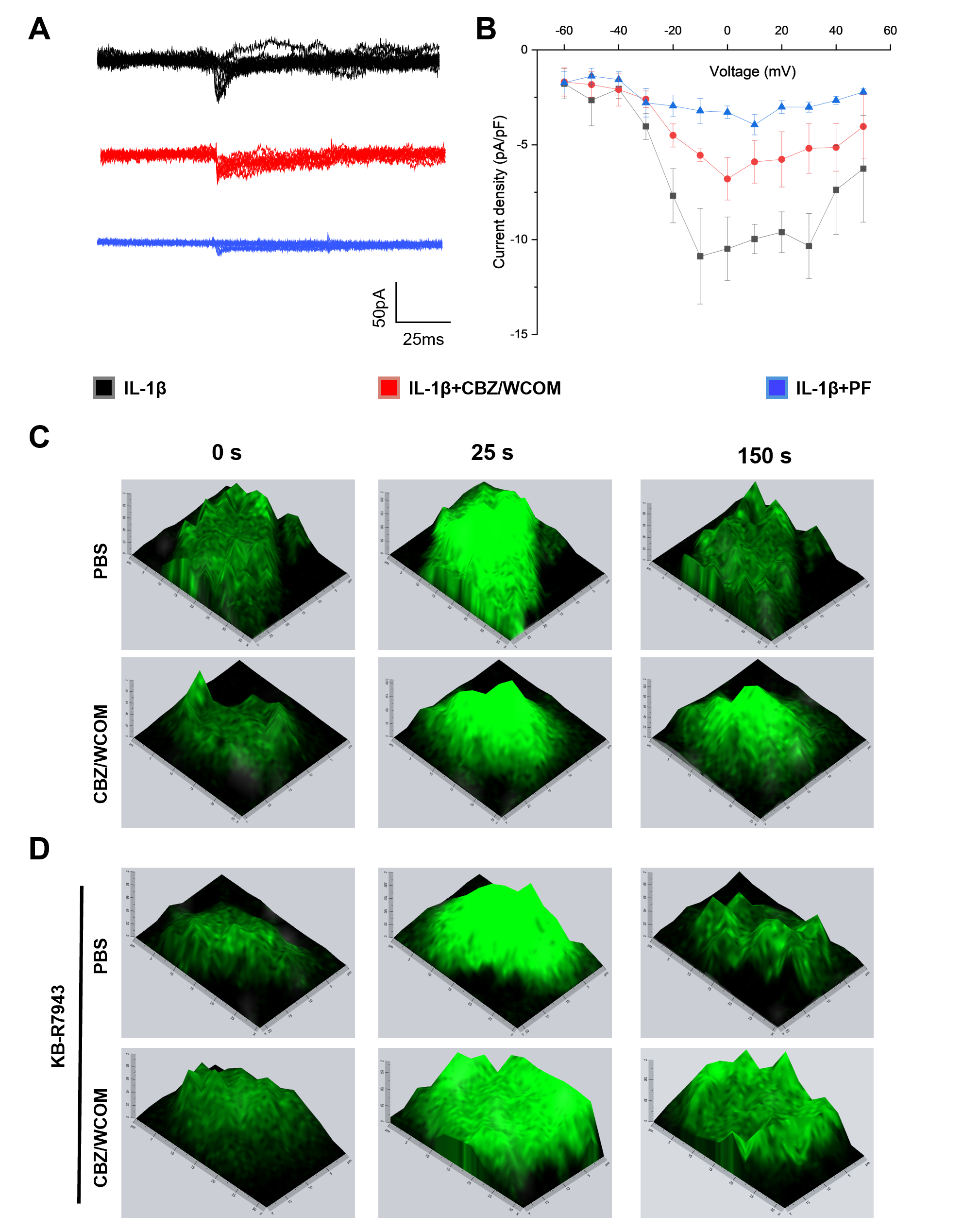
**

**Figure S4.** Supplementary electrophysiological and calcium-imaging analyses of Nav1.7-related sodium channel activity and NCX-associated Ca²⁺ regulation in chondrocytes. (A) Representative whole-cell sodium current traces recorded from chondrocytes in the IL-1β, IL-1β + CBZ/WCOM, and IL-1β + PF groups. (B) Current density–voltage (I–V) relationship curves in the IL-1β, IL-1β + CBZ/WCOM, and IL-1β + PF groups. (C) Representative intracellular Ca²⁺ imaging of chondrocytes in the PBS and CBZ/WCOM groups at different time points after ATP stimulation. (D) Representative intracellular Ca²⁺ imaging of chondrocytes in the PBS + KB-R7943 and CBZ/WCOM + KB-R7943 groups at different time points after ATP stimulation. For electrophysiological analyses in (A, B), n = 5. Representative Ca²⁺ imaging results in (C, D) are from three independent experiments.


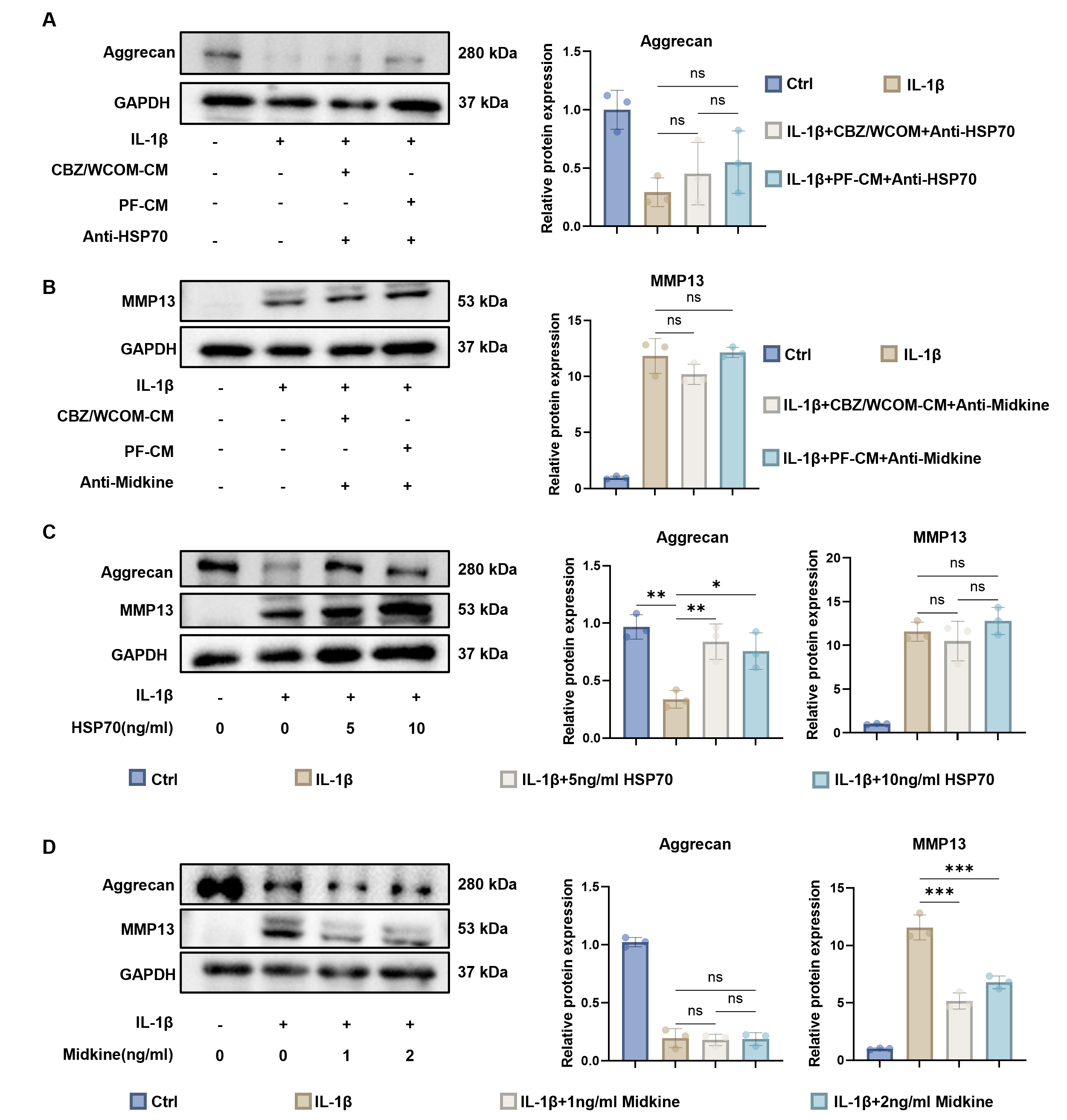


**Figure S5.** Neutralization and rescue assays showing the downstream involvement of HSP70 and Midkine in conditioned-medium-mediated regulation of chondrocyte metabolism. (A) Western blot analysis and densitometric quantification of MMP13 and Aggrecan expression in chondrocytes treated with conditioned media from CBZ/WCOM- or PF-treated chondrocytes after HSP70 neutralizing antibody treatment. (B) Western blot analysis and densitometric quantification of MMP13 and Aggrecan expression in chondrocytes treated with conditioned media from CBZ/WCOM- or PF-treated chondrocytes after Midkine neutralizing antibody treatment. (C) Western blot analysis and densitometric quantification of MMP13 and Aggrecan expression in chondrocytes treated with different concentrations of recombinant HSP70 protein. (D) Western blot analysis and densitometric quantification of MMP13 and Aggrecan expression in chondrocytes treated with different concentrations of recombinant Midkine protein. Data are presented as mean ± SD; n = 3. *, *p* < 0.05, **, *p* < 0.01, ***, *p* < 0.001.

**
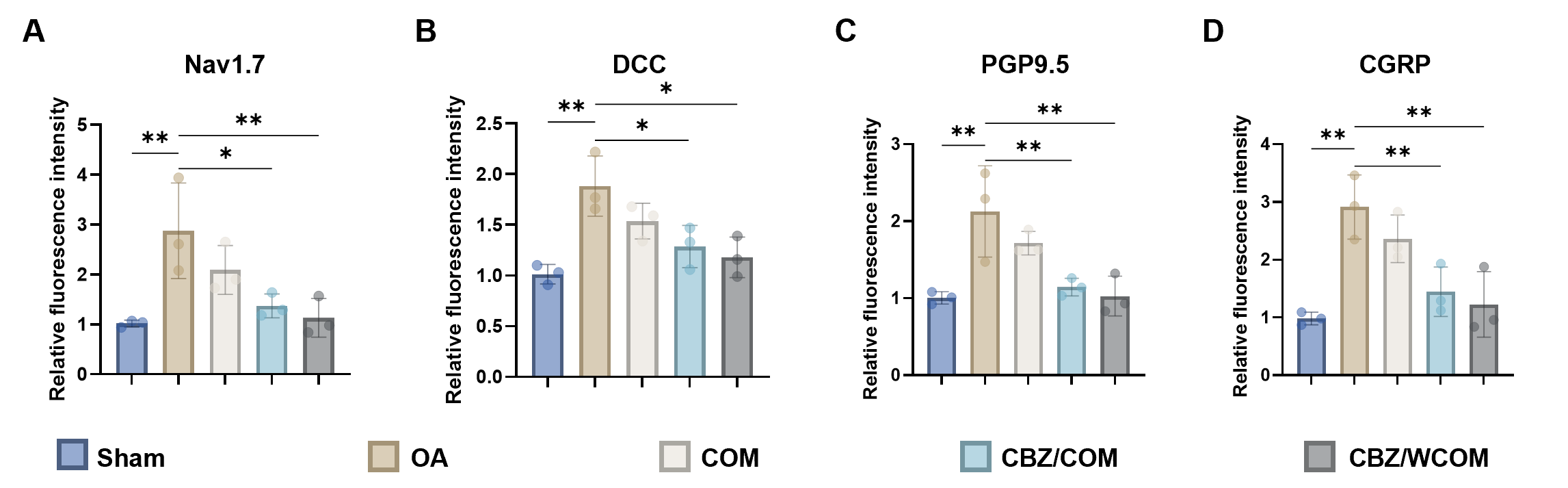
**

**Figure S6.** Quantification of pain-related ion channel and neural remodeling markers in DMM-induced osteoarthritis after intra-articular treatment with microspheres. (A) Quantification of Nav1.7 fluorescence intensity in knee joint sections from the indicated groups. (B) Quantification of DCC immunofluorescence intensity in the indicated groups. (C, D) Quantification of PGP9.5 and CGRP fluorescence intensity in the indicated groups. Data are presented as mean ± SD; n = 6, with three sections analyzed per mouse. *, *p* < 0.05, **, *p* < 0.01, ***, *p* < 0.001.


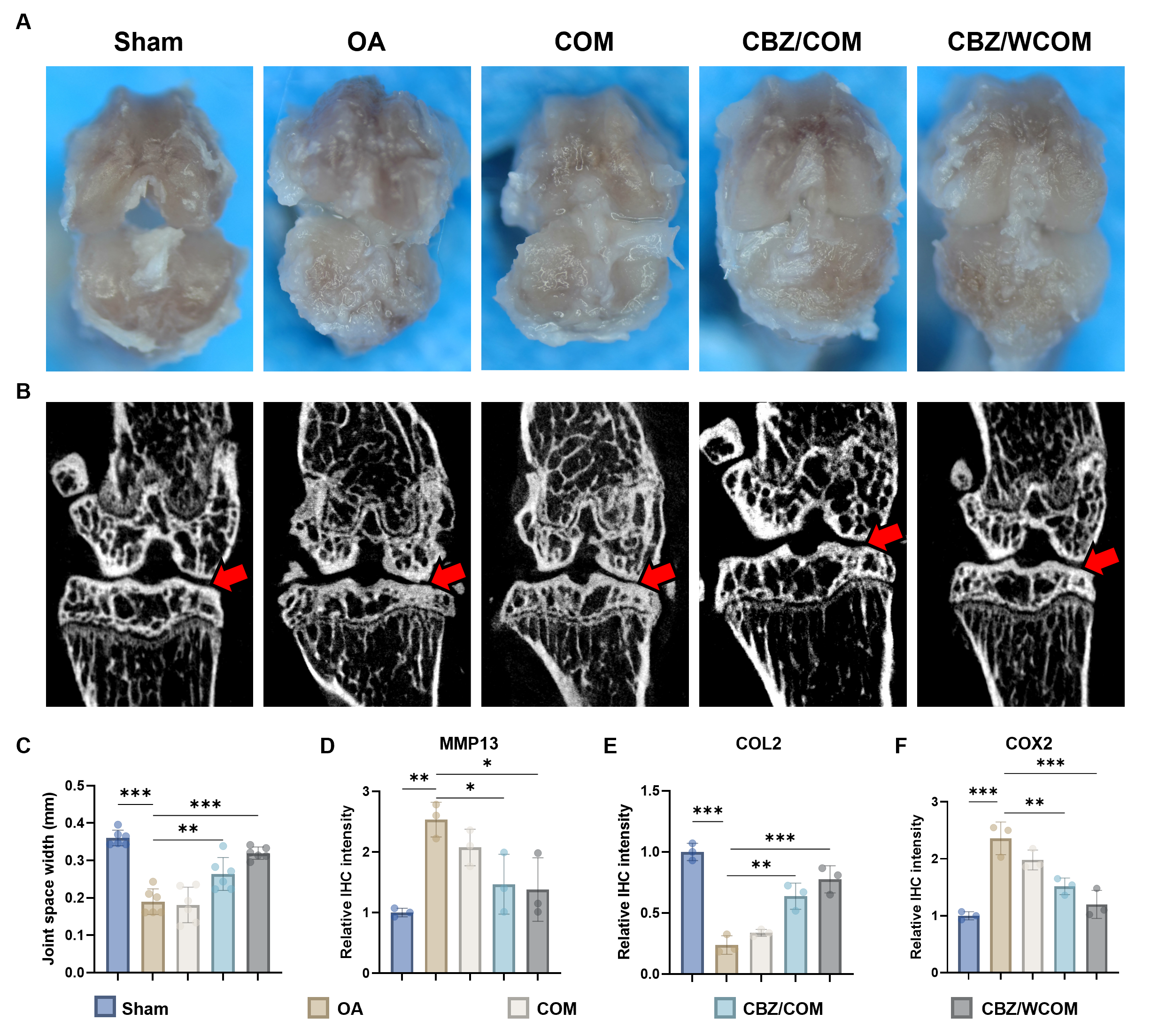


**Figure S7.** Macroscopic and micro-CT imaging of knee joint morphology and IHC quantification in DMM-induced osteoarthritis following intra-articular treatment with microspheres. (A) Representative gross morphology images of knee joint specimens from the indicated groups. (B) Representative micro-CT images of the medial compartment and subchondral bone region of interest. (C) Quantitative analysis of joint-space width in the indicated groups. (D–F) Quantification of MMP13, COL2, and COX2 immunohistochemical staining intensity in knee joint sections. Data are presented as mean ± SD; n = 6, with three sections analyzed per mouse for IHC quantification. *, *p* < 0.05, **, *p* < 0.01, ***, *p* < 0.001.


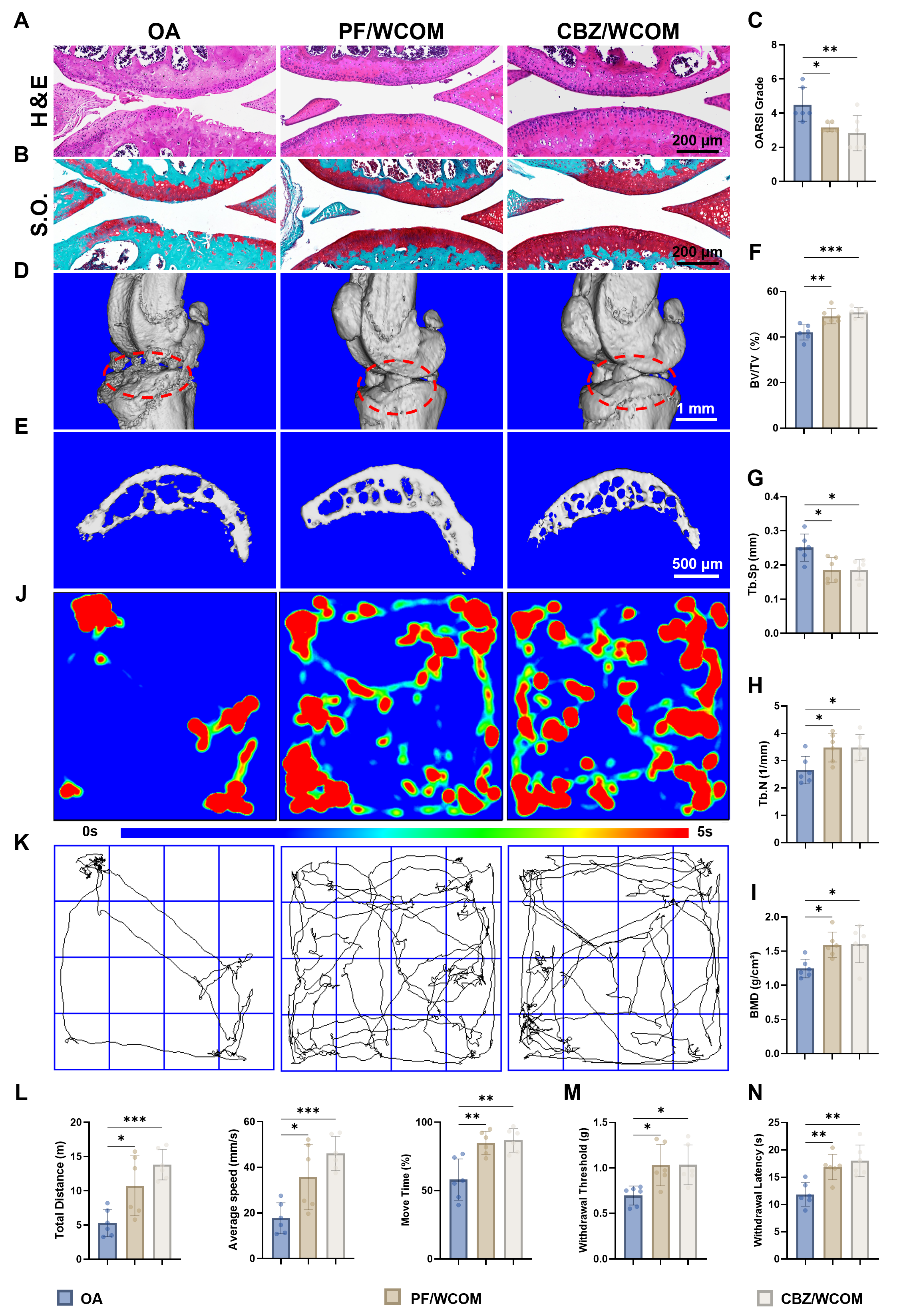


**Figure S8.** Supplementary in vivo validation of PF/WCOM and CBZ/WCOM in DMM-induced osteoarthritis. (A, B) Representative H&E and Safranin O–Fast Green staining images of knee joint sections from the OA, PF/WCOM, and CBZ/WCOM groups. (C) Quantitative analysis of cartilage degeneration using OARSI scores. (D) Representative three-dimensional micro-CT reconstructions of knee joints from the indicated groups. Red dashed circles indicate the joint region of interest. (E) Representative reconstructed images of subchondral bone. (F–I) Quantitative analysis of subchondral bone parameters, including BV/TV, Tb.Sp, Tb.N, and BMD, in the indicated groups. (J, K) Representative open-field heatmaps and movement trajectories in the OA, PF/WCOM, and CBZ/WCOM groups. (L) Quantification of open-field behavioral parameters, including total distance, mean velocity, and activity time ratio. (M, N) Evoked pain assessment by von Frey test and hot-plate test in the indicated groups. Data are presented as mean ± SD; n = 6. *, *p* < 0.05, **, *p* < 0.01, ***, *p* < 0.001.
